# Efficient siRNA delivery to murine melanoma cells *via* a novel genipin-based nano-polymer†

**DOI:** 10.1039/d4na00363b

**Published:** 2024-07-19

**Authors:** Giulia Della Pelle, Tim Bozic, Marija Vukomanović, Gregor Sersa, Bostjan Markelc, Nina Kostevšek

**Affiliations:** a Department for Nanostructured Materials, Jožef Stefan Institute 1000 Ljubljana Slovenia giulia.della.pelle@ijs.si; b Jožef Stefan International Postgraduate School 1000 Ljubljana Slovenia; c Department of Experimental Oncology, Institute of Oncology Ljubljana 1000 Ljubljana Slovenia; d Advanced Materials Department, Jožef Stefan Institute 1000 Ljubljana Slovenia; e Faculty of Health Sciences, University of Ljubljana Zdravstvena pot 5 SI-1000 Ljubljana Slovenia

## Abstract

Small-interfering RNAs (siRNAs) are therapeutic nucleic acids, often delivered *via* cationic polymers, liposomes, or extracellular vesicles, each method with its limitations. Genipin, a natural crosslinker for primary amines, was explored for siRNA delivery scaffolds. Spermine/genipin-based G*x*S5 polymers were synthesized, showing slightly positive *ζ* potential at neutral pH and intrinsic fluorescence. We then tuned their polymerization adding glycine to the reaction batch, from 1 to 10 molar ratio with genipin, therefore conferring them a “zwitterionic” character. G*x*S5 efficiently internalized into B16F10 murine melanoma cells, and exhibited strong siRNA-complexing ability and they were able to elicit up to 60% of gene knock-down without any toxicity. This highlights G*x*S5's potential as a safe, replicable, and tunable platform for therapeutic nucleic acid delivery, suggesting broader applications. This innovative approach not only sheds light on the intricate genipin reaction mechanism but also underscores the importance of fine-tuning nanoparticle properties for effective siRNA delivery. G*x*S5's success in mitigating cytotoxicity while maintaining delivery efficacy signifies a promising step towards safer and more efficient nucleic acid therapeutics.

## Abstract 2

A novel genipin and spermine based nanosized polymer exhibits excellent siRNA transfection ability and self-fluorescence properties. Size and surface charge can be tuned by glycine addition. The cytotoxicity was found to be remarkably low.

## Graphical abstract

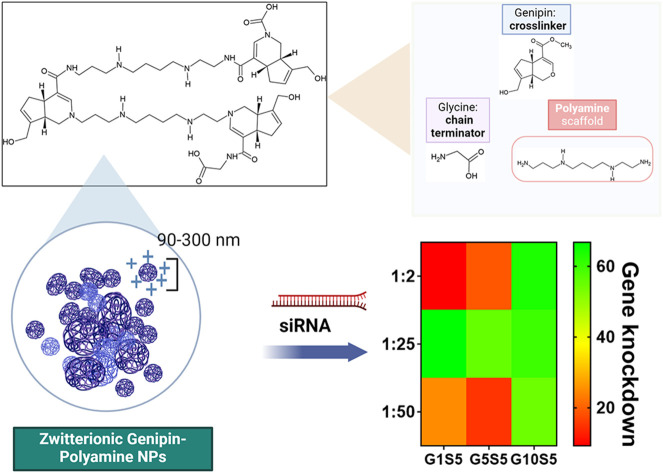

## 1 Introduction

The story of the clinical translation of small-interfering RNA (siRNA) began in 2018, with the approval of Patisaran.^1^ Due to their 3′ overhangs, small-interfering RNAs can elicit RNA interference upon RISC-complex binding, taking advantage of the pre-existing machinery originally destined for micro RNAs (miRNAs).^2^ Quantitatively speaking, the exact number of siRNA molecules per cell necessary to elicit a strong response varies according to a variety of factors: membrane permeability, internalization efficiency, and endosomal escape rate.^3^ Quantitatively speaking, the number of siRNA molecules per cell, for lipofectamine-siRNA complexes, was reported to be as low as few thousands of molecule per cell necessary to elicit a detectable knock-down, with a strict dependence on the sequence potency.^4^ Lipofectamine, although very effective, is known to be toxic.^4,5^ Therefore, the development of a safer alternative is of great interest. Endosomal escape still represents the major bottleneck to overcome in non-viral siRNA transfection:^6^ mammals do not possess any specific RNA transporter, in contrast to Nematoda species, such as *Caenorhabditis elegans*.^7,8^ Whereas single-stranded antisense oligonucleotides can be easily modified to be RNAse-resistant and are promptly endocytosed by cells, only 1% of the injected dose in mice, in the absence of coating or nano-encapsulation, spontaneously escapes the endosomes.^9^

In contrast, double-stranded, small-interfering RNAs cannot be chemically modified to a great extent. They are, therefore, readily degraded by circulating RNAses and by anti-viral machinery. A carrier is required to ensure a longer circulation time and an effective transfection.^10,11^ Most common approaches to siRNA delivery, such as lipofection or the use of polycations, take advantage of the well-established proton-sponge effect, and subsequent “endosomal escape”.^12^ For very positively charged carriers, though, it is safer to speak about “endosomal rupture” rather than “endosomal escape”. The lumen of an endosome, while maturing into a late endosome (pH 5–5.5), and, later, turning into a lysosome, is subjected to acidification by ATPase proton pumps. During the occurrence of this phenomenon, the presence of any ionizable conjugate or small molecule with H-buffering capability leads to an increase in the accumulation of Cl^−^ ions within the lumen (as a co-substrate of H^+^). Later, lysis of the membrane can occur, with the simultaneous release in the cytoplasm of an ionizable conjugate acting as a transfection agent – although resulting, briefly, in a certain degree of cytotoxicity.^13,14^ Therefore, the so-called, yet debated, proton-sponge effect,^15^ is connected to the buffering capability of the transfection agent, as N-quaternized PEI shows a reduced transfection ability.^16^ Now, an ideal cationic transfection agent should be able to escape the endosomes, causing a limited rupture. It must possess a moderate buffering capability to balance the delicate interplay among (a) a nucleic acid complexing ability, (b) cytotoxicity due to endosomal rupture, (c) active or passive internalization, (d) a residency time in the cytoplasm, and (e) an ability in the case of siRNAs to release the cargo for RISC-complex uptake. Apart from buffering capabilities, another essential characteristic of a polymeric transfection agent should be intrinsic in its spatial arrangement in aqueous medium. Upon the total protonation of amine groups, the monomer size should increase slightly due to the electrostatic repulsions,^17,18^ leading to an easier decomplexation of the payload.

A wide range of nanomaterials is currently available for small-interfering RNA transfection, mostly relying on artificial polycationic polymers such as PEI, PAMAM, block-copolymers or surface-engineered liposomes.^19–22^ Conventional dendrimer approaches suffer from a high-to-moderate cytotoxicity due to the concentrated positive charge, rapid clearance or kidney/liver bioaccumulation, lack of biodegradability *in vivo* and scarce solubility. Extreme cationic charge, indeed, despite representing the easiest route for a nanoparticle to enter a cell membrane, leads to a cascade of events, including Ca^2+^-induced apoptosis.^23,24^ Therefore, due to an increasing interest in nucleic acid delivery for gene therapy, the development of novel, safer and highly efficient transfection agents is vital to overcome the limitations of the current delivery systems. To reach that goal, a good balance among a number of factors must be achieved, *i.e.*, the ability to form complex therapeutic nucleic acids *via* either ionic bridges and hydrophobic interactions,^25^ the ionizability of amine groups within the maturing endosome compartment,^13^ and, finally, an adequate endosome-escape ratio without significantly impacting the cell viability by activating apoptotic pathways.

Here, we introduce novel, genipin-based, cationic polymer nanoparticles (NPs) as a safe and efficient RNA transfection agent of natural origin, which also has inherent fluorescence that can be used for imaging purposes. Our polymers are composed of polyamines, genipin and glycine. To the best our knowledge, this is the first time that such a combination has been used for the preparation of well-characterized and stable nanoparticles for the delivery of a nucleic acid.

Polyamines, *i.e.*, spermine and spermidine, were chosen because they are naturally complexed with mRNAs and other cytosolic RNAs.^26–28^ More specifically, spermine in solution inserts itself within the minor groove of double-stranded RNA,^27^ and it locks it in a relatively stable configuration that prevents aggregation. Then, genipin was applied to crosslink the polyamines. Genipin, a naturally occurring iridoid (belonging to the family of cyclic monoterpene secondary metabolites),^29^ has a relatively low cytotoxicity compared to other common crosslinkers and it can react with unprotonated amines, yielding products of various colours according to the amine moiety's chemical environment.^30^ Genipin-crosslinked polymers also exhibit red fluorescence,^31,32^ which can be exploited for intracellular tracking and the investigation of degradation pathways.

**Fig. 1:**
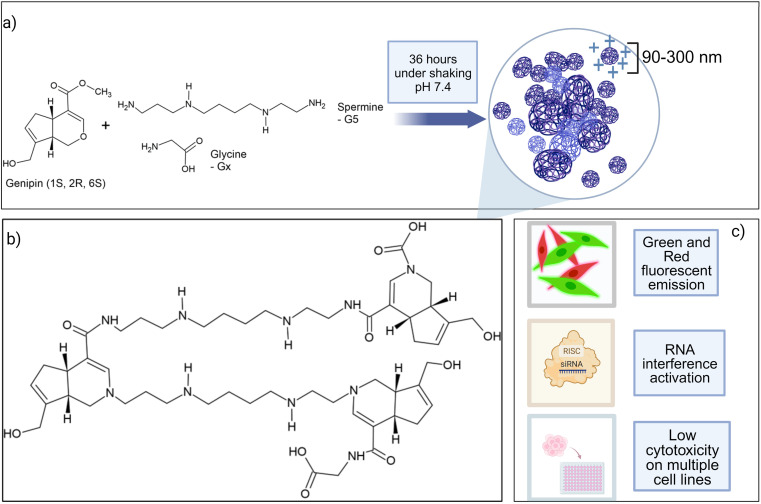
(a) Components of G*x*S5 polymers and overview of the synthesis process; (b) general structure of G*x*S5 polymers; (c) main properties of G*x*S5 polymers. For the sake of simplicity, some chemical bonds have been elongated and the image is not representative of the structural arrangement of the polymer in water or a buffer.

The genipin was allowed to react with different molar ratios of naturally occurring polyamines, spermine and spermidine. Glycine, a small amino acid with only one reactive amino group was chosen to terminate the polymerization chain of the genipin and spermine moieties. Finally, a 1 : 5 molar ratio of genipin-to-spermine was chosen, while the glycine molar ratio was kept variable. Polymers were therefore called G*x*S5, where “G” stands for glycine, “*x*” for the different molar ratios of glycine-to-genipin, “S” stands for spermine, whereas “5” stands for its molar ratio with regard to genipin (Fig. 1). We first characterized the genipin reaction mechanism and kinetics in the presence of spermine and spermidine in terms of the reaction rate and reaction order. Next, the fluorescence of the products and their anti-oxidant activity were quantitatively investigated. Their small-interfering RNA complexing ability, complex stability, cytotoxicity and behaviour under endosome-mimicking conditions were investigated for gene-silencing applications. Finally, the capability to transfect murine melanoma cells with an anti-reporter gene siRNA was demonstrated. The presence and the molar ratio of glycine was found to be instrumental with regard to the particle size, cytotoxicity, siRNA complexing ability, and transfection efficiency.

## 2 Results

### 2.1 UV/vis analysis of genipin–spermine-based polymers

**Fig. 2:**
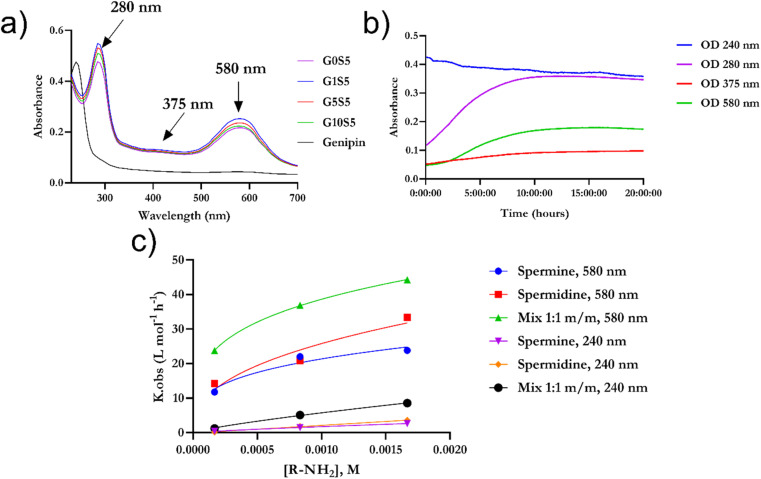
(a) Fig. 3 UV/vis spectra of genipin–polyamines–glycine polymers. The initial genipin concentration was set at 0.167 mM and it was consistent for the four samples. Final P580 concentration is 0.38 ± 2.5 10^−4^ and does not differ significantly among the formulations (*n* = 6 for each kind of sample). (b) Example of evolution in terms of absorbance of a genipin-polymerization reaction in the presence of spermine. For experiments regarding the nature of the mechanism and reaction kinetics of genipin, glycine was omitted. (c) *K*_obs_ calculated with genipin consumption monitored at 240 nm and 580 nm *vs.* amine concentration. Fitting was determined according to eqn (11) using data from Table 1.

Genipin is known to react with unprotonated primary amines,^33^ but its reaction kinetics with naturally occurring spermine and spermidine, each one of them possessing two primary amines, is yet to be fully investigated. First, the spectra of genipin–polyamine-based polymers showed the typical features of a crosslinked genipin matrix^34,35^ (Fig. 2a, and b). Due to the iridoid ring opening, genipin's peculiar peak at 240 nm evolves into three different signals at 290, 375 and 580 nm. Subsequently, the final polymerization product, with an absorbance peak at 580 nm, will be termed P580. Four tested molar ratios of genipin-to-glycine, defined as G*x*S5 (Materials and methods), gave rise to remarkably similar spectra, with maxima at 290 and 580 nm (Fig. 2a), and a weak hump at 375 nm. Then, to ascertain whether there was any significant difference in terms of reaction kinetics between the spermine and spermidine, different molar ratios of polyamines to genipin (as in Table 1) were incubated for 48 hours at room temperature, at pH 7.4, in the absence of glycine. Genipin concentration was kept constant at 0.000168 M. The absorbances at 240 nm, 280 nm, 375 nm and 580 nm were continuously monitored (Fig. 2b). The reaction was over after 36 hours of incubation (data not shown). The decrease, over time, of unreacted genipin was derived from a calibration curve (Fig. S1†). The increase in the absorbance of the intermediates at 280 nm and 375 nm is due to genipin consumption (240 nm) as well as the formation of the final product absorbing at 580 nm. The genipin consumption, monitored at 240 nm, and the formation of the final product at 580 nm were chosen to investigate the final kinetics law to shed light on its reaction mechanism, and allow us to optimize subsequent steps for our polymer's synthesis.

**Table 1:** Values obtained for *k*_obs_ for each concentration of polyamines and amine sources. Data were then used in the plot in Fig. 2c and fitted in eqn (11)

Amine source	[R–NH_2_] (M)	*K*_obs_ (M^−1^ h^−1^)	Monitored parameter (M)
Spermine	0.000167	11.78	[P580]
Spermidine	0.000167	14.22	[P580]
Spermine/spermidine 1 : 1	0.000167	23.76	[P580]
Spermine	0.00083	22.02	[P580]
Spermidine	0.00083	20.86	[P580]
Spermine/spermidine 1 : 1	0.00083	36.88	[P580]
Spermine	0.00167	23.80	[P580]
Spermidine	0.00167	33.44	[P580]
Spermine/spermidine 1 : 1	0.00167	44.28	[P580]
Spermine	0.000167	0.41	[Genipin]
Spermidine	0.000167	0.2	[Genipin]
Spermine/spermidine 1 : 1	0.000167	1.23	[Genipin]
Spermine	0.00083	1.46	[Genipin]
Spermidine	0.00083	1.82	[Genipin]
Spermine/spermidine 1 : 1	0.00083	5.12	[Genipin]
Spermine	0.00167	2.65	[Genipin]
Spermidine	0.00167	3.60	[Genipin]
Spermine/spermidine 1 : 1	0.00167	8.55	[Genipin]

Furthermore, to ascertain the reaction order relative to the two reagents, *i.e.*, genipin and the primary amine-group donors (the polyamines spermine and spermidine), we first evaluated the apparent consumption speed *ϑ*_*n*_ (M h^−1^) at 240 nm in terms of the genipin consumption (eqn (1)), where *n* stands for different genipin concentrations (*n*_1_–*n*_4_, see Table S1†).1
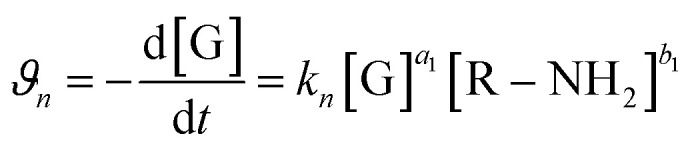


[G] and [R–NH_2_] are the concentrations of genipin and polyamine, respectively, with [R–NH_2_] being a constant (0.00083 M). *a*_1_ and *b*_1_ are the reaction orders and *k* is the kinetic constant. The isolation method was used to determine the reaction order *a*_1_ for genipin consumption. In the isolation method, one reagent is kept constant while the others are varied over a known range, and the initial reaction speeds *ϑ*_*n*_, defined as the slope of the linear area of the absorbance plot, are compared.^36^ Thus, different 
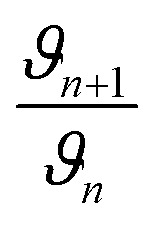
 ratios of the initial [G] concentrations were used, as shown in eqn (2). Finally, the Van't Hoff differential method^37^ was applied to derive *a*_1_ (eqn (3)). The reaction order for the consumption of genipin monitored at 240 nm was found to be *a*_1_ = 1.57 ± 0.25.2
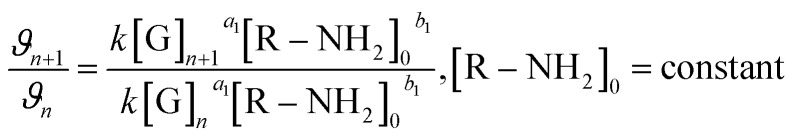
3
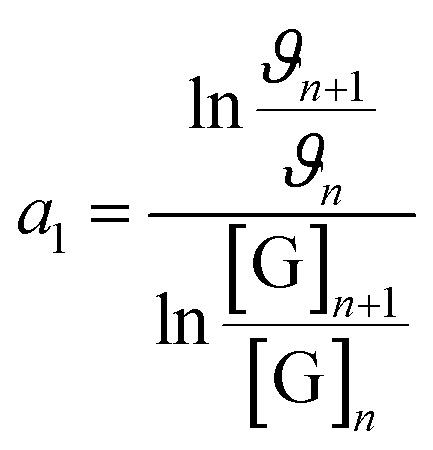


Then, the same protocol was applied to determine *b*_1_ for the three amine sources. Again, different 
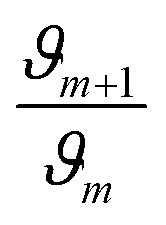
 ratio combinations of the initial [R–NH_2_] concentrations were used (see Table S2†) and [G] was kept constant at (0.000168 M) to derive eqn (4), which allows the calculation of *b*_1_. The three amine sources [R–NH_2_] (spermine, spermidine, and 1 : 1 m m^−1^ mix of them) were mathematically treated separately (see Table S2†). The reaction order was calculated to be *b*_1_ = 1.16 ± 0.32. For further analysis, we approximate *a*_1_ and *b*_1_ to be 1.5 and 1, respectively.4
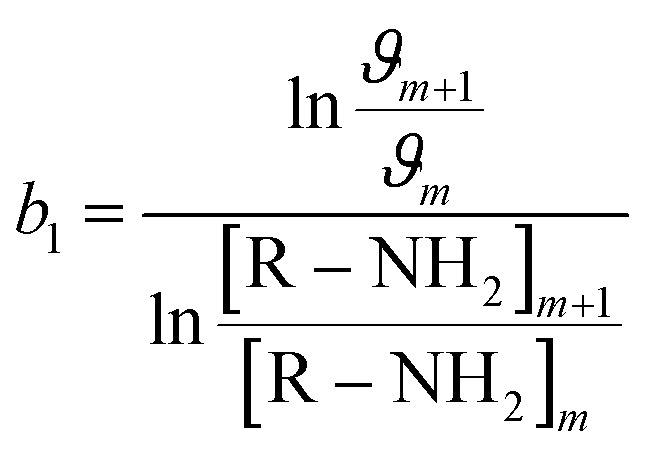


The same protocol was applied to determine the reaction orders *a*_2_ and *b*_2_ for the formation of the product absorbing at 580 nm, termed P580 (Tables S3 and S4†), expressed as eqn (5):5
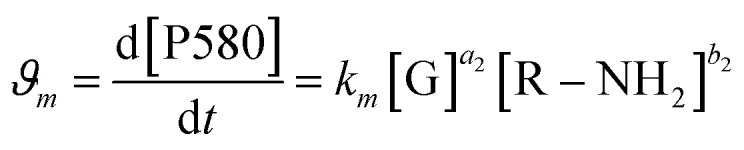


For the formation of the product, reaction orders *a*_2_ and *b*_2_ were found to be 1.51 ± 0.8, with respect to genipin, and 0.53 ± 0.17, with respect to the concentration of the amine groups. We approximate their values to be *a*_1_ = *a*_2_ ≈ 1.5 and *b*_2_ ≈ 0.5.

Using different initial concentrations of genipin also allowed us to calculate the molar extinction coefficient *ε* of P580 (*ε*_P580_ = 8.5 10^3^ mol^−1^ L^−1^), the product appearing at 580 nm, as the slope of the plot in Fig. S2a,† and a path-length correction protocol was used. *ε* was used to estimate the P580 concentration (Fig. S2b†) in later experiments.

At this stage of the analysis, the reaction laws, for the consumption of genipin and the production of P580, respectively, can be approximated as:6
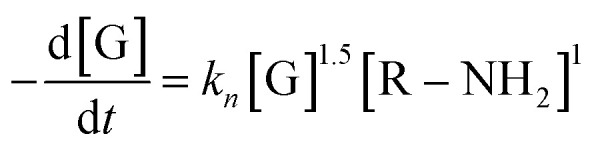
7
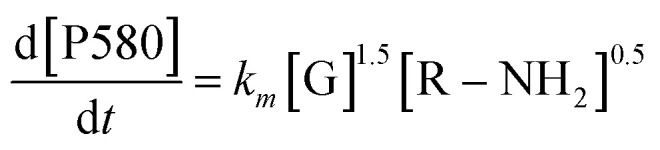


To determine the rate-limiting step of the genipin polymerization and shed more light on its reactivity within our system, the reaction-rate constants *k* must be determined. The values can be obtained by solving the following equation:*K*_obs_*m*,*n*__ = *k*_*n*,*m*_[R–NH_2_]^*b*_1,2_^where *k*_obs_ is generally defined as the observed experimental rate constant, dependent on the initial concentration of the unisolated reactant, *i.e.*, primary amines, the reaction step, and the monitored parameter. Therefore, pseudo-order reactions as mathematical treatments must be introduced, using the integration method described by Upadhyay.^36^ To calculate the observed reaction-rate constant (*k*_obs_) we used the methodology presented in ref. 36 for the fractional reaction orders *b*_2_ = 0.5 and *a*_1_ = 1.5 (Fig. S3†). By integrating eqn (6) and (7) over time, respectively, for all concentrations of [G] and [P580], we obtained the following, as generalized forms:8
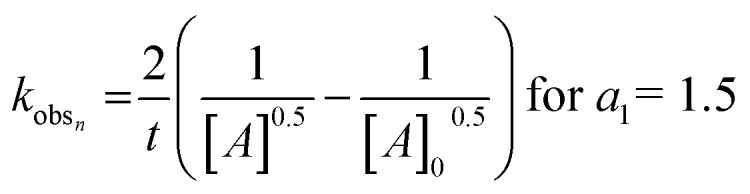
9
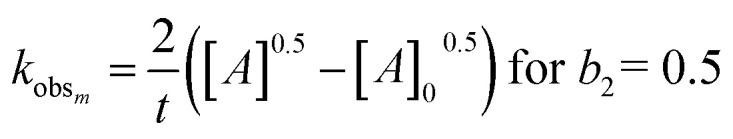
where [A] is the concentration of genipin or P580 at any given time, and [A]_0_ is the initial concentration.

Again, as formalized by Upadhyay, to obtain the value of the observed experimental reaction constants *k*_obs_, from eqn (8) and (9) we can extract and plot 1/[G]^0.5^ or 1/[P580]^0.5^ against time (Fig. S3a and S3b,† respectively, expressed in hours). As a derivation from eqn (8) and (9), the values of *k*_obs_ are equal to twice the slopes (eqn (10)). Each amine group source (spermine, spermidine, and 1 : 1 mix of them) was mathematically treated separately (Table 1 and Fig. 1c). The slopes of the plots were calculated and the *k*_obs_ values were obtained with the *r*^2^ > 0.97.

10 *k*_obs_ = 2 × slope

For the genipin consumption monitored at 240 nm, the experimental reaction order of *b*_1_, 1.16 ± 0.32 was confirmed to be approximately 1, from the least-squares fitting of eqn (11) (0.81 ± 0.06, 1.054 ± 0.02, and 0.79 ± 0.09 for spermine, spermidine, and 1 : 1 molar mix), and the rate constant *k*, L mol^−2^ h^−2^ was subsequently derived using the already-introduced relationship (Fig. 2c):

11 K_obs_*n*,*m*__ = *k*_*n*,*m*_[R–NH_2_]^*b*_1,2_^

where *k*_obs_*m*,*n*__ are the observed experimental reaction constants, *k*_*n*,*m*_ are the reaction constants for the genipin consumption and P580 formation, and *b*_1,2_ are the reaction orders obtained with the exponential fitting (*r* > 0.95). The reaction constants *k*_*n*_ for the consumption of genipin under our conditions are 153.3 ± 9.12, 968.7 ± 12.64, and 507.5 ± 34.12 L mol^−2^ h^−2^ for the three amine sources: spermine, spermidine, and 1 : 1 molar mix, respectively. The 1 : 1 mix's kinetic reaction constant was found to be between those of spermine and spermidine. Applying the same procedure for the formation of P580, the experimental reaction order *b*_2_ with regard to the amines for the formation of the P580 (0.53 ± 0.17, Table S3†) was confirmed *via* the fitting of eqn (6) (*r*^2^ > 0.97), obtaining a value of *b*_2_ equal to 0.43 ± 0.04, 0.51 ± 0.09, and 0.49 ± 0.07 for spermine, spermidine, and the 1 : 1 molar mix, respectively (Fig. 2c). The reaction-rate constants *k*_*m*_ for the formation of P580 were found to be 105.8 ± 7.34, 275.5 ± 63.9, and 204.8 ± 46.5 L mol^−2^ h^−2^ for spermine, spermidine, and the 1 : 1 molar mix, respectively, fitting eqn (11). Therefore, the final crosslinking of the genipin moieties, represented by the formation of P580, is the limiting step of the reaction, as reported in ref. 33 and 38, *i.e.*, that dictating the actual reaction speed.

The final kinetic law of genipin polymerization in the presence of the amine sources is therefore:12
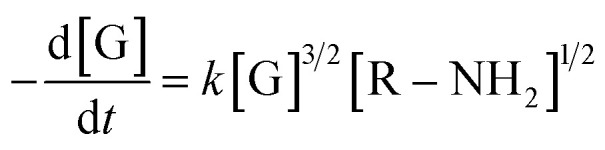


The values of the rate constants arise from the charge distribution within the polyamine molecule. Indeed, the p*K*_a_ of the two external amines of spermine are respectively 10.9 and 10.1, while those of spermidine 10.1 and 9.9,^39^ thus increasing slightly (0.11% of spermine *vs.* 0.25% of spermidine) the chances of a primary amine not being protonated and therefore available for reaction.

Despite this finding, we decided to choose spermine as the primary amine source for the synthesis of our polymer due to its longer aliphatic chain and higher net-positive charge.

### 2.2 Size, zeta potential and ultrastructure characterization of genipin–polyamine-based polymers: optimization of molar ratios of the polyamines

**Fig. 3:**
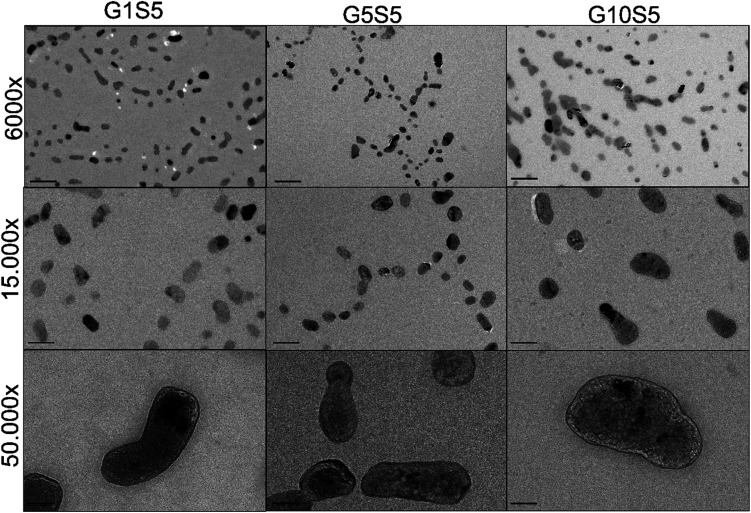
TEM pictures of genipin-based polymers. From top to bottom, the scalebar is, respectively, 500, 200 and 50 nm. The magnification is reported on the left side.

The dynamic light-scattering technique was used to investigate the size and *ζ* potential of our polymers. We first determined which polyamine, *i.e.*, spermine and spermidine, was more suitable as a scaffold (Table S5†). The spermidine-based polymers had a lower *ζ*-potential (5–12 mV) than the spermine-based polymers (12–18 mV), likely due to the protonation of the only remaining amine in the aliphatic chain not occupied in crosslinking with genipin, in place of spermine having two amines that can sport a positive charge. Within the same molar ratio range for genipin (1–10), no improvement in this parameter for the genipin/spermine molar ratios higher than five was observed. Spermine's extra internal amine group therefore provided a higher surface positive charge and, probably, a higher nucleic-acid-complexing ability. For this reason, we decided to keep genipin/spermine 1 : 5 as the molar ratio for all future synthesis (Table 2), while changing the glycine molar ratio. Since glycine possesses only one amine group, such an amino acid was chosen to end the polymerization chain, tentatively reducing the size of the nanoparticles and conferring on them a zwitterionic character. Since genipin is only able to react with unprotonated amine groups,^40^ and the glycine amine group p*K*_a_ is 9.8 at 25 °C, roughly only about 1% of all the groups would be available for a reaction at pH 7.4 at any moment. For all the spermine/glycine combinations the *ζ* potential was found to be positive (from +18.1 to +12.53 mV, Table 2), and the glycine molar ratio had a moderate effect on the value, decreasing it from G1S5 to G10S5. Furthermore, the increasing glycine concentration influenced the nanoparticles' size. At a 1 : 10 molar ratio with genipin, the particle size was found to be less than 200 nm for most of the recorded events. All three polymers showed a majority, in terms of the percentage of recorded events, of small nanoparticles (100–300 nm) and a minority of aggregates ranging from 800 nm to a micron. The two main particle populations were, respectively, termed “Population 1” (P1) and “Population 2” (P2). To characterize the polymers' ultrastructure, we conducted an aspect-ratio analysis using TEM images (Fig. S4,†*n* of images = 10). Confirming the DLS data, several larger clots can be observed among an overwhelming majority of smaller particles. For the sake of simplicity, we focused on smaller particles (Population 1). The polymers appeared as dark, mostly regular, ellipsoidal clots that rapidly disintegrated under the TEM's electron beam (Fig. 3, 50.000 magnification). The glycine's increasing molar ratio proved to be instrumental in the formation and stabilization of individual nanoparticles, since no well-dispersed particle can be noted in the case of G0S5 (Fig. S4†). Surprisingly, the aspect ratio of G10S5 was higher than for G5S5 and G1S5(Fig. S4†). While G1S5 and G5S5 were found to follow a lognormal distribution, the G10S5 aspect-ratio frequency distribution was ambiguous, with only a Kolmogorov–Smirnov test considering it (*p* = 0.035) lognormal. Moreover, while the G1S5 and G5S5 aspect-ratio distributions are not significantly different, a Kruskall–Wallis test proved that the G10S5 nanoparticles' dimensions statistically diverge from the others (*p* = 0.005 with regard to G5S5 and *p* = 0.0007 with regard to G1S5). The apparent radius of a circle with the same area as the ellipse was used to compare the P1's and P2's relative abundancies in the TEM images. The latter yielded similar results to that already obtained with the DLS, confirming the reliability of this technique for such an object. As already known,^41^ DLS overestimates the particle size by around 1.5 to 2 times due to the hydration-shell disturbance. Finally, we found a linear relationship between the mass concentration of the G*x*S5 polymers and the OD at 580 nm, which was used in all the subsequent experiments to calculate the mass concentration of the samples. The protocol and calibration curve are shown in the ESI (Fig. S5).†

**Table 2:** Comparison of *ζ*-potential, DLS (number-weighted) and TEM particle size for different spermine-based polymer formulations, ±SD. The ratio between the two populations (see text for definition) is reported as %. DLS data arise from five independent measurements for each formulation of three different technical replicates. TEM particle size measurements were obtained with the ImageJ built-in measuring tool, on the basis of *n* = 10 micrographies for each formulation, three technical replicates of each G*x*S5, at 15.000× magnification

Sample, molar ratio with genipin	ζ potential, mV	DLS particle size (nm)	Ratio P1/P2% in DLS	PDI (×100)	TEM diameter, nm	Ratio P1/P2% in TEM
Genipin: glycine: spermine = 1 : 1 : 5 (G1S5)	18.1 ± 2.6	271.3 ± 49.5	1.23 ± 0.67	15.23	160 ± 20	5.02 ± 1.78
		832.4 ± 275.5		11.17	948 ± 12	
Genipin: glycine: spermine = 1 : 5 : 5 (G1S5)	12.5 ± 1.5	190.04 ± 72.7	3.86 ± 0.69	2.76	125 ± 16	9.47 ± 2.36
		1187.4 ± 683		6.06	1065 ± 50	
Genipin: glycine: spermine = 1 : 10 : 5 (G10S5)	12.3 ± 0.7	120.6 ± 10.7	5.93 ± 1.54	7.94	98 ± 28	14.36 ± 3.61
		997.8 ± 23.5		5.33	576 ± 73	
Genipin: glycine: spermine = 1 : 0 : 5 (G0S5)	18.9 ± 1.1	1014.3 ± 400	∼0	15.46	Not recognizable individual nanoparticles	

### 2.3 Fluorescence investigation of genipin–spermine–glycine-based polymers

**Fig. 4:**
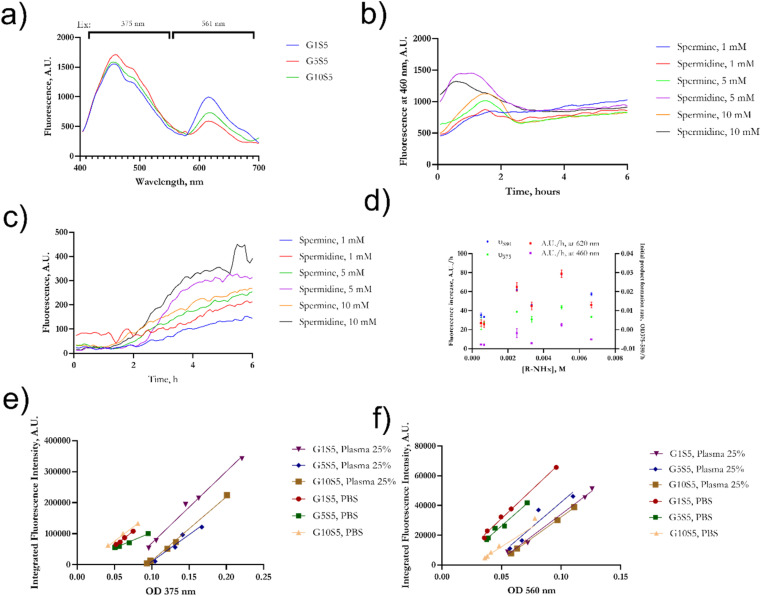
(a) Merged emission spectra of column-purified polymers recorded at two different excitation wavelengths. (b) Kinetics of fluorescence emission for 375/460 nm ex/em; (c) for 561/615 nm ex/em. For the kinetic studies, no glycine was added, and the chosen amine source was spermine. (d) Rate of increase of fluorescence/hour, within 4 hours – linear range, compared with P580 formation rate and 375 nm product formation. (e) Plots of OD at 375 m *vs.* the integrated fluorescence intensity between 425 and 600 nm. (f) Plots of OD at 375 m *vs.* the integrated fluorescence intensity between 580 and 700 nm. Data (±SD) in (b–d) refer to the same experiment, *n* = 3 technical replicates.

Although the fluorescence properties of genipin products are already known,^32,40^ they were never investigated in a quantitative manner and in the presence of aliphatic amine sources such as spermine and spermidine. Two excitation/emission peaks were identified (Fig. 4a) when the polymers were dissolved in PBS, one with a near-UV excitation maximum at 375 nm, and emitting in the green region, and another in the blue area (561 nm), while emitting in the near red. Then, we monitored the evolution of the two fluorescence peaks over 6 hours at 375/460 nm em/ex and 561/620 em/ex (Fig. 4b, and 4c). Such timing was chosen to investigate the earlier, rate-determining hours of the reaction (Fig. 4b).

Interestingly, the evolution kinetics in terms of the A.U./h of the peak recorded in the red at 620 nm (Fig. 4c, and e) was found to be related (correlation matrix, *p* < 0.001) to the formation of the product at 580 nm (see Section 2.1), hinting that such a fluorescence emission is due to the final crosslinked genipin moieties, previously termed P580. On the other hand (Fig. 4b, and d), the nature of the peak being excited in the near-UV (375/460 ex/em) and emitting in green looks to be correlated (correlation matrix, *p* = 0.002) with the formation of an intermediate, weakly absorbing at 375 nm (Fig. 2a, and b). The fluorescence intensity of such an intermediate appears to rapidly reach a peak and then start to steeply decrease during the first two hours of reaction as already observed in ref. 38, therefore purportedly originating from an amine-substituted genipin. Such an intermediate disappears over time due to its final crosslinking.

We also tested whether the fluorescence emission was concentration-dependent and if any self-quenching effect was noticeable in the P580 molar-concentration range between 0.2 mM and 5 μM. We monitored the signal at em/ex 375/460 and 560/620 nm. The plots of [P580] against fluorescence emission were found to be linearly correlated for both maxima (Fig. S6†), with *r*^2^ > 0.90 in all cases. Nevertheless, deviations from linearity appear evident from [P580] > 0.00012 M, hinting at a self-quenching at high concentrations (Fig. S6†).

#### 2.3.1 Quantum-yield (QY) determination in water and human plasma

We then calculated the fluorescence quantum yield of both peaks (*Φ*_f_) of the G*x*S5 polymers using the gradient method.^42,43^ Fluorescein isothiocyanate (FITC) was used as a reference dye for the green emission maximum, and rhodamine B was used for the red. Optical data for both dyes were obtained from ref. 44. Quantum yields *Φ* were calculated according to the equation:13
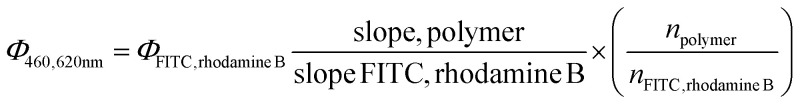


Meanwhile, the “slope” is defined as the slope of the linear plot for the OD at the excitation wavelength *vs.* the integral of the emission spectrum of the dye excited at that wavelength, and *n* is the refractive index of the solvent. ODs between 0.01 and 0.1 were used for the reference dyes, while P580 molar concentration was kept below 0.00012 M to avoid self-quenching effects and therefore variations from linearity. Slopes of reference dyes did not significantly differ from PBS to plasma 25% v/v (ANCOVA test, *p* = 0.59 for FITC and *p* = 0.29 for rhodamine B, Fig. S7b†). Both peaks of the polymers reflected a relatively low QY compared to rhodamine B (36%)^45^ and FITC (90%),^46^ as reported in Table 3, in an aqueous environment, while moderately increasing in the presence of plasma proteins. When mixed with plasma, the emission peak of all the polymers (Fig. S7†) was superimposed to that of the NADH ring: indeed, in plasma, two prominent peaks can be seen (see Fig. S7†), around 445 nm and the other one at 485 nm, belonging to the nicotinamide ring^47^ present in NADH, one of the main fluorescence emitters in plasma. In the presence of plasma proteins, the QY slightly increased in all three polymers both in the red and green regions, but not in a statistically significant fashion (*p* > 0.2 for all buffer *vs.* plasma comparisons). The slopes of G*x*S5 appear substantially parallel, with no significant variations due to glycine concentration in the reaction batch (Fig. 4e, and f). The calculated blueshift in 25% plasma *vs.* water of the emission peak accounted for 15 nm with all three polymers.

**Table 3:** Quantum yield of G*x*S5 polymers relative to reference dyes, expressed in %

Polymer	H_2_O, 375 ex/460 em	Plasma 25%, 375 ex/460 em (M^−1^)	H_2_O, 561 ex/620 em	Plasma, 25% v/v, 561 ex/620 em
G1S5	0.55 ± 0.004	2.01 ± 0.011	0.76 ± 0.025	0.98 ± 0.007
G5S5	0.52 ± 0.002	1.50 ± 0.010	0.62 ± 0.023	1.01 ± 0.011
G10S5	0.67 ± 0.009	0.95 ± 0.012	0.47 ± 0.036	0.89 ± 0.007

### 2.4 Reaction with ninhydrin for amine-group determination

The number of amine groups available for nucleic acid complexation is a crucial parameter for the successful transfection of therapeutic nucleic acids. A ninhydrin assay is a widely used test for determining the concentration of amine groups,^48^ and, although it has been extensively used for such a determination in chitosan,^49^ extracellular matrix^50^ and collagen^51^ crosslinked substrates, to the best of our knowledge, this is the first reported use of such an assay for genipin–polyamine-based polymeric nanoparticles. It is known that the ninhydrin absorbance spectrum shows different peaks according to the various amine sources the molecule is complexed with.^52^ In our case, the main ninhydrin peak at 570 nm, conventionally used for quantification, overlaps with the P580 broad absorbance peak of the polymers (Fig. S8a†), and the signal at 400 nm was absent, whereas it was present when spermine is used as the amine source for a standard curve. Therefore, we checked the reliability of the signal present at 345 nm, while the concentration of [R–NH*x*], with a signal at 570 nm, was calculated by subtracting the polymer background. We used spermine as the source of amines for the calibration curve, determined at 345 nm (*r*^2^ 0.97) and 570 nm (*r*^2^ 0.92). Due to the low molar absorptivity at 570 nm of the complex formed by ninhydrin and polymer, we also used the signal at 345 nm for amine-group determination. The final molar concentrations of the amine groups for 100 mg ml^−1^ polymers are reported in Table 4.

**Table 4:** Concentration of amine groups in G*x*S5 polymers rehydrated at 100 mg ml^−1^ according to ninhydrin and TNBS assays. The ratio of amines detected with TNBS assay *vs.* ninhydrin assay is reported

Sample	[R–NH*x*] at 345 nm, ninhydrin assay, M	[R–NH*x*], at 570 nm	[R–NH_2_], TNBS assay, M	TNBS/ninhydrin assay detected [R–NH*x*]
G1S5	0.123 ± 0.002	0.148 ± 0.054	0.029 ± 0.002	0.21
G5S5	0.155 ± 0.003	0.163 ± 0.033	0.038 ± 0.002	0.24
G10S5	0.182 ± 0.006	0.181 ± 0.011	0.048 ± 0.002	0.26

To ascertain the reliability of the ninhydrin assay in quantifying the secondary amines, we also carried out a TNBS assay (Table 4), which is widely reported as being able to form absorbing complexes only with primary amines,^53^ using glycine as the standard reference (Fig. S8b†). Indeed, the ninhydrin assay was capable of detecting almost four times the amount of amines of TNBS.

Subsequently, concentrations obtained from the ninhydrin assay were used to calculate the total amine concentration, and it will be referred to as total nitrogen amount, *N*, from now on. As reported in ref. 52, only proline and hydroxyproline have a signal maximum at 350 nm, followed by a faint hump at 400 nm, indicating the presence of *cis*-oriented secondary amines. The ninhydrin assay is therefore a valid assay to determine the concentration of the secondary and primary amine groups in this novel class of polymeric nanoparticles.

### 2.5 Genipin-based polymers and their ability to complex with small-interfering RNA in polyplexes: optimization of siRNA phosphate/total nitrogen (P/N) ratio for tdTomato knock-down

#### 2.5.1 *ζ* Potential and particle size investigation

**Fig. 5:**
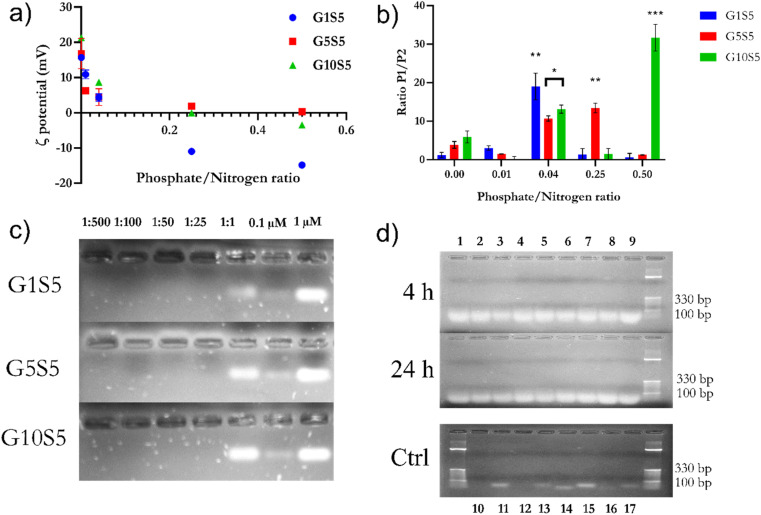
(a) Evolution of *ζ* potential ±SD in mV according to the P/N ratio for the three polymers. Data refer to 3 independent technical replicates for each G*x*S5, coming from the same synthesis batch. at pH 7.4. (b) Ratio of number of recorded events as “Population 1” and “Population 2”, whereas P2 is assumed to be resulting from the aggregation of nanoparticles from P1, against siRNA backbone phosphates/polymer nitrogen ratio. The relative abundance of P1 was assessed to be statistically different from the titration starting point (in the absence of phosphate backbone groups) with ANOVA two-ways. Data refer to 3 independent technical replicates, each one composed of 10 measurements. G*x*S5 came from the same synthesis batch. (c) Agarose gel migration of different P/N ratios of polymers and siRNA. For comparison, two different concentrations of free siRNA were added (0.1 and 1 μM) (d) RNase A (5 μg ml^−1^) and heparin displacement assay. Lanes are as follows: (1) G1S5, P/N 1 : 2; (2) G1S5, P/N 1 : 25; (3) G1S5, P/N 1 : 50; (4) G5S5, P/N 1 : 2; (5) G5S5, P/N 1 : 25; (6) G5S5, P/N 1 : 50; (7) G10S5, P/N 1 : 2; (8) G1S5, P/N 1 : 25; (9) G10S5, P/N 1 : 50; (10) naked siRNA 1 μM, RNase A+, 24 h; (11) naked siRNA 1 μM, RNase A−, 24 h; (12) naked siRNA 0.1 μM, RNase A+, 24 h; (13) naked siRNA 0.1 μM, RNase A−, 24 h; (14) naked siRNA 1 μM, RNase A+, 4 h; (15) naked siRNA 1 μM, RNase A−, 4 h; (16) naked siRNA 0.1 μM, RNase A+, 4 h; (17) naked siRNA 0.1 μM, RNase A−, 4 h.

Due to their polymeric nature and positive charge, the G*x*S5 materials were expected to be able to complex nucleic acids. We titrated a fixed amount of polymer with increasing concentrations of small-interfering RNA (siRNA) against tdTomato fluorescent protein, thereby slowly increasing the molar phosphate-to-nitrogen (P/N) ratio, to simulate actual experimental conditions and avoiding sequence-related unexpected results. Upon addition of the siRNA, at neutral pH, the *ζ* potential drops until a negative plateau (Fig. 5a), −15, ≈ 0, and −2.5 mV mV for G1S5, G5S5, G10S5, respectively. We then aimed to optimize the phosphate-to-nitrogen ratios to be used in the knock-down experiment, in terms of particle size and nanoparticle aggregation upon siRNA titration (Fig. 5b). The response varies according to the glycine-to-genipin molar ratio in the initial reaction batch. In all three samples, a bimodal particle size distribution can be noted, which is due to the aggregation of smaller particles in response to the complexation with siRNA, with the population composed of larger aggregates ranging from 300 nm to 1 micron in diameter (termed “Population 2”, as in Ultrastructure and particle size of G*x*S5 polymers) increasing in size according to the siRNA concentration, whereas the population composed of smaller than 300 nm nanoparticles shows a less dramatic size increase (Population 1, Fig. S10†). These aggregates are stable over time in PBS 7.4 (data not shown). Although the presence of two particle size populations was already established *via* TEM on free polymers (Table 2), this increase in particle size can be directly addressed to a complexation with siRNA phosphate groups. The transition to Population 2 can be attributed to aggregation/flocculation due to the neutralization of repulsive charges and the complexation with siRNA, which increases the total bulk size (refer to Fig. S10† for size evolution). However, the ratio of the frequency of events relative to Population 1 or Population 2 does not follow a linear trend upon siRNA titration (Fig. 5b), but some areas of dramatic prevalence for Population 1 events can be noted. At a 1 : 25 P/N (0.04) ratio, polyplexes arising from the three G*x*S5 show a small and monodisperse particle size distribution. Interestingly, the higher the glycine molar ratio, the more significant the contribution by carboxylic groups belonging to genipin: electrostatic repulsions with phosphate groups probably cause an internal rearrangement within the genipin–spermine net, exposing more charged amine groups on the outside shell of the nanoparticle and thus increasing the surface available for siRNA complexing.

#### 2.5.2 Protection against RNase A degradation and intercalating dye exclusion

To ascertain the intercalating dye-exclusion ability of the three polymers, different P/N ratios (1 : 500 to 1 : 1) were diluted in PBS with a pH of 7.4 and incubated with 1 μM of anti-tdTomato siRNA in 10 μl of PBS. Subsequently, polyplex samples were run on a 1.5% agarose gel pre-stained with Midori Green. No signal could be detected in the lanes containing the siRNA/G*x*S5 complexes up to a 1 : 1 ratio, suggesting the impossibility of Midori Green being able to bind to nucleic acid due to highly tight complexation (Fig. 5c) with the polymers, and, subsequently, the lack of fluorescence. Although present at a 1 : 1 P/N ratio, the band belonging to the complexes appears to be delayed in migration, as reported already elsewhere.^22,54,55^

Furthermore, the protection ability against RNAse A degradation was also assayed. Briefly, siRNA polyplexes of 1 : 25 and 1 : 50 P/N ratios of G*x*S5 polymers were incubated with 5 μg ml^−1^ of RNase A for 4 and 24 hours (Fig. 5d). Subsequently, the polyplexes were de-complexed with heparin. All three polymers offered excellent protection against siRNA degradation, while lanes containing free siRNA incubated with the enzyme showed no band, even after 4 hours.

#### 2.5.3 G*x*S5 behaviour in an endosomal-mimicking environment and potentiometric back-titration

**Fig. 6:**
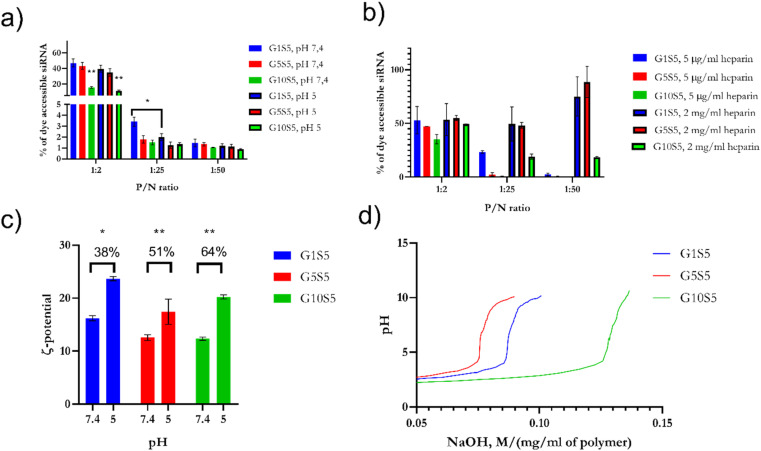
(a) Spontaneous release at pH 5 and pH 7.4 of siRNA from G*x*S5 polymers. siRNA de-complexation, measured as an increase or decrease of Quantus dye fluorescence, after incubation at pH 5 and pH 7.4. (b) To assess the relative strength of the complexation, samples were treated with heparin at increasing concentrations of heparin for pH 5 and pH 7.4. (c) Change in *ζ* potential of GxS5 from pH 7.4 to 5; data were collected over 5 replicates for each pH/polymer combination. (d) Back-titration of G*x*S5 with NaOH 0.1 N, normalized for the dilution of the polymer. *p* ≤ 0.05*, *p* ≤ 0.01**, *p* ≤ 0.001***, *p* ≤ 0.0001****.

Endosomal pH being mildly acidic,^17,56^ amine groups are expected to be fully protonated, with unforeseeable consequences regarding polyplex de/stabilization. Therefore, we first tested the avidity of the G*x*S5 polymer in complexing with siRNA, pre-incubated with Quantus dye, detected as the decrease of fluorescence due to dye exclusion. Subsequently, we monitored the eventual spontaneous release of the siRNA from polyplexes, G1S5, G5S5 and G10S5 at 1 : 2, 1 : 25 and 1 : 50 P/N ratios, at pH 7.4 and pH 5, during 3 hours of incubation and 37 °C. Finally, we assayed the relative complexation strength, by de-complexing siRNA–G*x*S5 polymers by increasing the amounts of heparin, measured as the increase in the baseline fluorescence, due to the re-accessibility of the dye to the substrate. G10S5 exhibited the highest dye exclusion of the three polymers at both pH values compared to G1S5 and G5S5 (Fig. 6a, *p* = 0.0037). The behaviour of the polymers at mildly acidic pH, *i.e.*, the slightly increased complexation with siRNA, suggests further protonation, with G1S5 being the polymer with the highest further complexation at pH 5, at 1 : 25 P/N (*p* = 0.021). In the presence of heparin, G1S5 at 1 : 25 P/N had already released roughly 50% of siRNA at 5 μg ml^−1^, while G10S5 released a similar amount at only 2 mg ml^−1^ of heparin (Fig. 6b). Overall, G10S5 showed the highest siRNA retention and the most stable complexes, while, intuitively, the higher the P/N ratio, the lower the complexation strength. The *ζ*-potential data confirmed that G10S5 has the highest increase in protonation among the three polymers, with a 64% increase in surface charge at lower pH (Fig. 6c, ANOVA one-way). Interestingly, upon acidification, the ratios between Population 1 and Population 2 of the nanoparticles rose significantly, with a dramatic shift towards Population 1 (assumed to be composed of monomers of the polymer, 100–300 nm, Table S6†), with a substantial percentage increase in the monomer particle size. While the first effect can be related to stronger electrostatic repulsions due to further amino-group protonation, surprisingly, the highest growth in particle size was shown by G10S5: from a roughly 2.25-fold increase in terms of diameter, the volume of the monomers increased more than eleven times, suggesting an additional endosomal evasion mechanism (Table S6†).

To further investigate the re-protonation phenomenon in G*x*S5, we performed a back titration (Fig. 6d) with NaOH from pH 2 to 10. G1S5 showed a lower buffering capability than G5S5 and G10S5. The latter showed a more extended buffering ability due to the larger number of carboxylic acid moieties belonging to glycine. At the same time, the tertiary amines' p*K*_a_ were found to be, respectively, for G1S5, G5S5, and G10S5, 7.16, 7.09 and 6.57. Therefore, the lower p*K*_a_ in the case of G10S5 explains the higher degree of further complexation with siRNA at lower pH and eventually a more substantial proton-sponge effect.

### 2.6 Cytotoxicity of G*x*S5 polymers on different cell lines

**Fig. 7:**
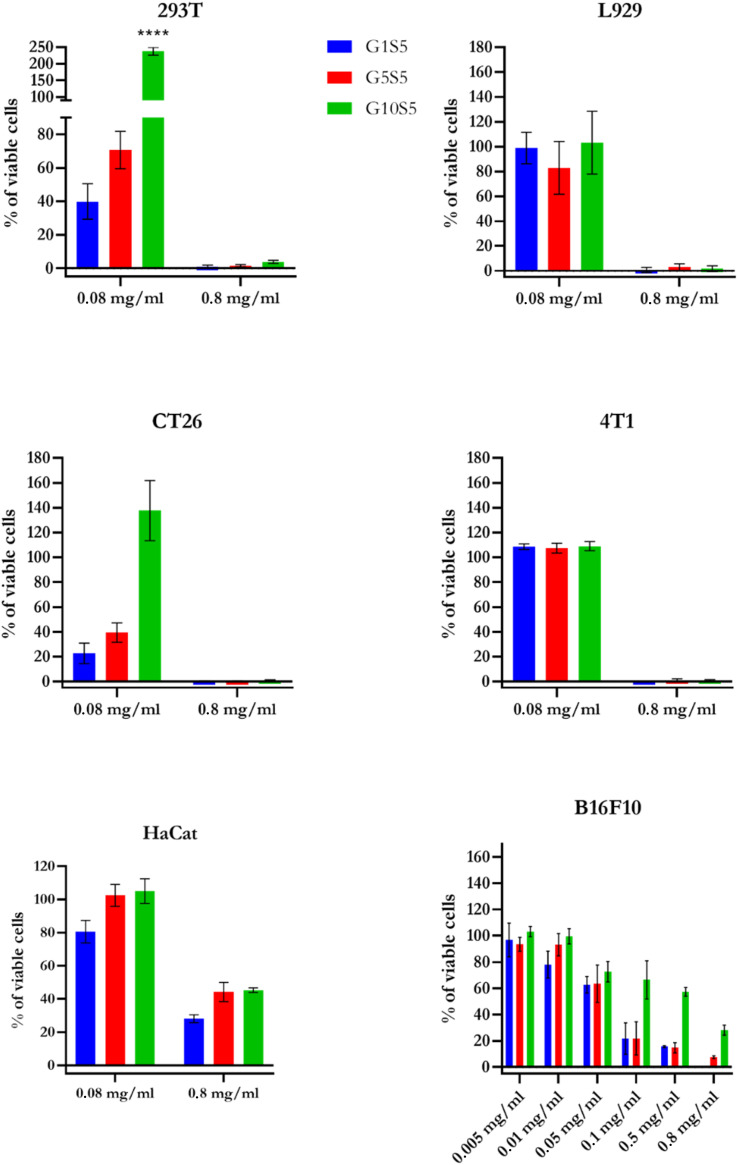
Presto Blue detected cytotoxicity at increasing concentrations of G*x*S5. HaCat data are relative to 6 days of co-incubation. For all cell lines except for HaCaT, the incubation time was 72 hours. Data (*n* = 6 for every concentration/cell line) are ±SD. *p* ≤ 0.0001****.

To evaluate the toxicity of the polymeric nanoparticles, several cell lines were incubated with different concentrations of G*x*S5 (Fig. 7a). Regarding the non-malignant cell lines, on kidney epithelial cells (HEK 293T) and murine connective-tissue cells (L-929), G10S5 was the least toxic polymer at 0.08 mg ml^−1^, even though, at varying degrees, all the G*x*S5 were cytotoxic at 0.8 mg ml^−1^. Interestingly, G10S5 had a significant stimulating effect (*p* < 0.0001) on the growth of the HEK 293T cells.

G10S5 proved to be the least toxic polymer on the CT26 cells, while on the 4T1 breast-cancer model, at 0.08 mg ml^−1^, no polymer showed significant cytotoxicity. Furthermore, we tested the biocompatibility of our polymers on human immortalized keratinocytes (HaCaT). Even after 72 hours, the G*x*S5 polymers showed little-to-no toxicity on HaCaT cells (Fig. S12†). We therefore decided to wash the cells and replace the medium, and prolong the experiment for another 72 hours. After a total of 6 days of co-incubation, the resulting cytotoxicity followed the already-described trend, decreasing with the higher glycine molar ratio. Finally, on our cell line of choice for the knock-down experiments, B16F10, a wider range of concentrations was tested. Again, at the highest concentration (0.8 mg ml^−1^), only cells treated with G10S5 retained 20% of viability, following the already-described trend. Also, all the G*x*S5 polymers showed little-to-no hemolytic activity over fresh human red-blood cells (Fig. S11†), up to 0.02 mg ml^−1^, while lipofectamine 2000 was reported to have 20% of hemolytic activity at similar concentrations.^57^

### 2.7 Internalization of polymers and polymer/siRNA complexes in B16F10 cells and knock-down ability

**Fig. 8:**
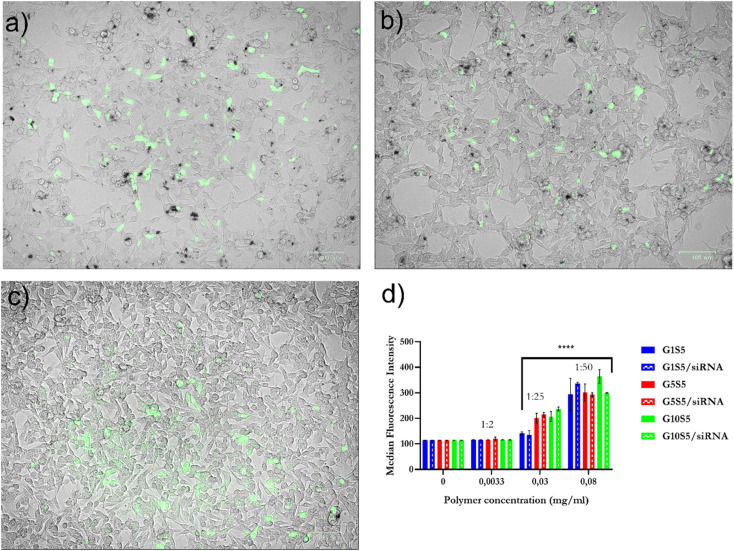
(a) B16F10 incubated with 0.08 mg ml^−1^ of G1S5, after 48 hours; (b) with G5S5; (c) with G10S5. Brightfield pictures are superimposed with FITC-channel acquisition. (d) Median Fluorescence Intensity of G*x*S5, complexed or not with siRNA, as detected with FACS in the PE channel (±SD, *n* = 3), *p* ≤ 0.0001****. The P/N ratio of polyplexes is reported above the plot.

To assess the internalization ratio of the G*x*S5 we took advantage of the intrinsic polymer fluorescence and measured it in the PE channel (ex: 560 nm, em: 585/15 nm) of the flow cytometer to detect their presence in B16F10 cells. The normalized median fluorescence intensity (MFI) of the live-cell population is related to the tested polymer concentration (Fig. 8d). Furthermore, the determined MFI did not change significantly when the cells were incubated with polymer/siRNA polyplexes, indicating that the complexation with siRNA, and, therefore, the lower *ζ* potential, did not significantly affect the internalization efficiency (Fig. 8d, and 5a for *ζ*-potential values). Also, despite their relatively low quantum yield, it was possible to visualize the internalization of the G*x*S5 in the FITC channel by B16F10 cells (Fig. 8a–c).

### 2.8 Efficacy as siRNA carrier for RNA interference

**Fig. 9:**
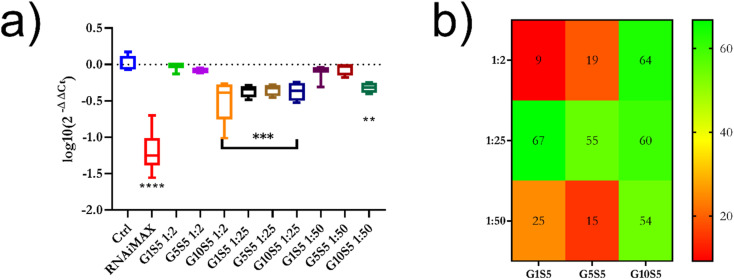
(a) Log 10 of tdTomato gene expression fold change, after transfection with 30 nanomoles of siRNA complexed with G*x*S5. Anova One-Way was used to assess the statistical consistency of the data. *p* ≤ 0.05*, *p* ≤ 0.01**, *p* ≤ 0.001***, *p* ≤ 0.0001****. (b) Heatmap with % of gene knockdown, according to polymer identity and P/N ratio.

The three G*x*S5 polymers, complexed with siRNA at different phosphate-to-nitrogen (P/N) ratios (1 : 2, 1 : 25 and 1 : 50) were tested for their ability to deliver siRNA targeting tdTomato to B16F10 cytoplasm and elicit RNA interfering towards the target gene (Fig. 8b–d). In all cases the polymer concentration in the wells was below the cytotoxicity threshold (Table S7† and Fig. 7e). As a preliminary study we used flow cytometry to determine the tdTomato fluorescence intensity at 72 hours after transfection with the polymer/siRNA complexes at a 1 : 25 P/N ratio (Fig. S13†). This ratio was chosen (Fig. 6b) due to the polymers showing an overwhelming majority of monodispersed, small, unclustered nanoparticles. All three tested polymer/siRNA polyplexes reduced the tdTomato MFI in the transfected cells compared to the control cells; however, their silencing efficacy was lower than RNAiMAX, which served as a positive control (Fig. S13†). As a proof of concept, within the same experiment, we also checked the capability of G0S5 – a polymer composed only of genipin and spermine – to transfect the B16F10 cells with siRNA targeting tdTomato (Fig. S13†). Interestingly, no silencing of the tdTomato was observed in the presence of the G0S5/siRNA complexes, indicating that the nanoparticulate and well-disperse nature of the G*x*S5 polymers is key to a successful transfection. Due to the overlapping of the tdTomato emission peak with the emission peak of our polymers, we used qRT-PCR to determine the tdTomato silencing efficacy of the polymer/siRNA complexes. We also tested two other P/N ratios, *i.e.*, 1 : 2 and 1 : 50, to investigate the effects of different nanoparticles' *ζ* potentials, from neutral to negative, on the transfection efficiency. All the polymers at a 1 : 25 P/N ratio showed around 60% of gene knockdown (Fig. 9a and b), while only G10S5 was effective at 1 : 2 and 1 : 50 P/N as well exhibiting 63% and 54% tdTomato knock-down, respectively. Empty polymers, at the same concentration of polymer/siRNA complex per well, did not significantly affect either the β-actin or the tdTomato gene expression (Fig. S14†).

## 3 Discussion

Genipin has been widely used as an ingredient in novel, nanoparticle-based delivery systems, but, to the best of our knowledge, this is the first reported therapeutic nucleic acid application. So far, genipin-based nanoparticulate delivery systems involved mostly topical applications: a 2003 breakthrough paper by Liang *et al.*^58^ demonstrated how genipin-crosslinked gelatin microspheres induce an inflammatory response in skeletal rat muscle, dramatically lower than glutaraldehyde, and, thus, are safer for *in vivo* application. A poloxamer–genipin-based hydrogel was also proven safe for intra-ocular quercetin delivery,^59^ while a lysine–folic-acid, genipin-based particulate dispersion was an efficient dyeing and doxorubicin-carrying agent for glioma cells.^60^

We thoroughly investigated with UV/vis and fluorescence spectroscopies the reaction kinetics of genipin incubated at pH 7.4 and in an excess of molecular oxygen with spermidine, spermine, and a 1 : 1 molar mix of both. Genipin reacts with overhanging primary amines of polyamines, very much depending on the pH, with a complex reaction mechanism involving at least two steps, *i.e.*, iridoid ring opening and heterocyclic ring formation, together with the simultaneous release of methanol and slow, rate-determining final crosslinking on an amidic moiety. The reaction orders are reported to be fractionary, and our findings therefore confirm the literature.^33,38,61^ According to ref. 33, genipin follows a second-order reaction in the presence of a primary amine source (*i.e.*, ethylendiamine), while we found it to follow instead an order of 1.5 for genipin consumption, with the final product appearing at 580 nm, and a square-root dependence on the amine groups' concentration, better reflecting the intricate mechanism of the reaction of genipin. Although already described,^32,40^ the fluorescence of genipin products was never thoroughly investigated and compared with well-known dyes. Our G*x*S5 polymers show fluorescence in the red area, as already well-established for genipin products,^62^ but the wider, green emitting maximum has never been investigated before. As reported in ref. 63, despite their excitation maximum being set at 480 nm and so only partially overlapping with our green signal, a net decrease follows an initial steep increase, also consistent with the kinetics of the peak appearing at 375 nm in our case. Such a signal is due to the heterocyclic pyridine ring of genipin.^38^ This result is consistent with the use of a single stereo-isomer source, as we did, and not with a racemic mixture. Pizzolitto *et al.*^38^ reported the computed UV/vis intermediate spectra of all three stereo-isomers of genipin and our results are consistent with the optical behaviour of *S*,*R*,*S* stereo-isomers. The green fluorescence emission of a racemic genipin mixture, if existing, is yet to be determined. Despite being relatively low, the fluorescence quantum yield in red G*x*S5 showed a slight improvement in the presence of serum proteins and it was strong enough to be detected for FACS, making it possible to follow, in further experiments, the route of the nanoparticles within the cells.

Since, in our case, tertiary amines are formed during iridoid-ring reactions with spermine and with the only amine of glycine, the higher the molar fraction of the latter, the higher the amount of tertiary amines in the polymer net, together with carboxylic moieties (Fig. 1). It has been widely demonstrated^17,64,65^ that the presence of ionizable tertiary amines is of crucial importance for successful and harmless gene delivery, since highly branched polymers sporting tertiary amines undergo, during endosome maturation and a subsequent decrease in pH, complete protonation and a net charge increase, ultimately leading to rupture of the endosome. For this reason, similar to our G*x*S5, histidine, due to its ionizable tertiary amine present in the imidazole ring, is a promising substituent in bio-compatible transfection agents.^66^ Our results in an endosomal mimicking environment confirmed the ionization of tertiary amine present on the indole ring of genipin, a destabilization of the nanoplex structure, as already reported,^17^ with a moderate but detectable increase in size due to electrostatic repulsions among the positively charged groups. G10S5 proved to be the polymer with the higher re-ionizability, the higher volume increase at acidic pH (instrumental, according to “the umbrella hypothesis”^67^), but, counterintuitively, also the faintest release in the presence of heparin (a negatively charged displacing agent), whereas G5S5 and G1S5 allowed the complete recovery of the siRNA at all the P/N ratios (Fig. 6a and b). On the other hand, at neutral pH, polymers, at any phosphate-to-nitrogen ratio, equally protected the siRNA from RNase A degradation and excluded siRNA-intercalating dyes in agarose gel up to a 1 : 1 P/N ratio. It must be pointed out that the condensation strength in polyplexes is the key to successful transfection: a too densely packed polyplex would be incapable of releasing the payload in the cytoplasm^68^ (or, if ever, it would happen after very long incubation times), it will cause excess cytotoxicity due to cationic charges.^69,70^ For instance, a reported threshold value for PEI is N/P > 3, since a certain number of free cationic chains is instrumental for plasmid transfection efficiency.^71^ In contrast, a too laxly packed one will release the payload in the culture medium, or it will not fully protect it from endosomal RNase/DNase degradation.^70^ The growing volumetric expansion from G1S5 to G10S5 upon acidification hints at an internal rearrangement, where the yet-unprotonated imidazole ring, upon protonation, comes into contact with the particle surface; the more pronounced this effect is, the purportedly higher is the transfection efficiency.

We then decided to focus on an often-overlooked parameter in therapeutic nucleic acid (TNA) transfection, such as the phosphate-to-nitrogen (P/N) ratio. The complexation between a cationic nanoparticle and TNA predictably changes the spatial arrangement of the nanoparticles, and such folding is connected to copies of the TNA per nanoparticle. In our case, this affected both the particle size and the *ζ* potential of the G*x*S5 nanosized polymers (Fig. 6c and S10†). At a 1 : 50 P/N ratio, or 0.08 mg ml^−1^ of concentration, G1S5 and G5S5 yielded little-to-no gene knockdown despite showing a high internalization efficiency *via* FACS and 80–90% of cell viability. This can be explained with the extremely tight complexation of the polymer and siRNA in high excess of unshielded cationic charges; the polyplexes, despite being effectively internalized, will not release their cargo for Argonaute uptake within the 48 hours of our experiment. All three polymer monomers at 1 : 50 P/N were less than 200 nm in diameter (Fig. S10†), with the average size of the G10S5 being 95 nm. G10S5, indeed, at a 1 : 50 P/N ratio, yielded an almost 60% of tdTomato knockdown: therefore, either the internalization mechanism followed by the latter must differ from the others due to a smaller size (*via* clathrin-mediated endocytosis, for instance), or the polymer shedding from siRNA, due to the shorter length of the genipin–spermine backbone and the presence of carboxylic groups, is more likely to happen.^72^

At a 1 : 25 P/N ratio, all the G*x*S5 showed a comparable, sustained gene knockdown and similar uptake efficiency in FACS: the behaviour of the polymers within endosomal mimicking experiments is consistent with such an outcome since all the polyplexes showed a well-monodispersed size distribution, a moderately positive (≈10 mV) *ζ* potential, a total dye exclusion in agarose gel and a 20–50% payload release upon the addition of 2 mg ml^−1^ of heparin, confirming every existing model about cationic-assisted transfection.^73,74^

Interestingly, only G10S5 showed a good knock-down effect at a 1 : 2 ratio (or 0.003 mg ml^−1^), as well: the siRNA/G10S5 complex possesses a neutral-to-negative surface charge and the highest, among the set, complexation strength (lowest release upon heparin incubation). Mott *et al.*^75,76^ recently demonstrated that, in HeLa and 293T cells, negatively charged polyplexes mostly co-localize with clathrin-coated vesicles, suggesting a different internalization pathway for G10S5 at a 1 : 2 P/N ratio in B16F10. We can, therefore, speculate that each polymer monomer can carry more than one copy of the siRNA since even at low concentrations, G10S5 can effectively deliver its payload to the cytoplasm. With only such a low amount required, there is room to scale up the concentration of polyplexes, while remaining within the high cell-viability window. The presence of a carboxylic group is instrumental to balancing good endosomal escape/direct internalization ratio with an effective condensation of siRNA: in the G*x*S5 polymers the supposed presence of ionic bonds between the carboxylic and amine groups within the polymer net helps create a tri-dimensional structure with better transfection-efficiency ability. Our overall data confirm that a transfection polymer's “zwitterionic” character plays a significant role in cell uptake, endosomal escape, and eventually successful transfection.^77,78^ Finally, a substantial role for G*x*S5 aggregates in successful transfection (“Population 2” in DLS and TEM, 0.3–0.9 μm of diameter) cannot be ruled out: as macropinocytosis was determined to be inducible in B16F10 cells,^79^ and, although the authors did not state the average molecular weight of the biopolymer, characteristic membrane ruffles were observed in confocal laser scanning microscopy. The latter are believed to form macro-pinosomes, intracellular vesicles exploited by several viruses to enter the cells,^80^ but their fate, whether they fuse with mature lysosomes or whether they are driven back to the plasma membrane, therefore releasing their cargo intracellularly, is yet to be determined, despite having been successfully exploited for nucleic acid delivery.^81^ It is also interesting how the zwitterionic character impacted the cytotoxicity on different cell lines. Our data overwhelmingly suggest that the lower, and more distributed, positive net charge significantly reduced the cytotoxicity. For comparison, Grecka *et al.*^82^ reported 80% B16F10 mortality in the presence of 0.01 mg ml^−1^ of PEI. Finally, as established by our results on fresh human erythrocytes, with little-to-absent lysis (Fig. S11†), while a nanoparticle–membrane interaction cannot be ruled out, any toxicity must be attributed to intracellular metabolic activity rather than simple membrane disruption.^83^

## 4 Conclusions

We developed a set of self-fluorescent, genipin–spermine-based polymers with a nucleic acid complexing ability and mild cationic charge. We succeeded in tailoring their size by changing the glycine molar ratio in the initial reaction batch, conferring on them a “zwitterionic” character, and improving their biocompatibility. Our data, taken together, suggest that genipin-based cationic polymers are a novel class of transfection agents. Their low cytotoxicity, mild reaction conditions, high tunability, good transfection efficiencies, and self-fluorescence properties make these polymers a platform suitable for the development of theranostic agents. In the future, we aim to elucidate their 3D-spatial arrangements *via* nuclear magnetic resonance, their *in vivo* and *in vitro* biodegradability, bioactivity, and clearance routes, and we aim to improve their transfection efficiencies *via* stealth coating or further conjugation.

## 5 Materials and methods

### 5.1 Reagents

For all purposes, DEPC Milli-Q water was used. Spermine–HCl (306-67-2), spermidine (124-20-9), glycine (56-40-6), heparin sodium salt (porcine intestinal mucosa, 9041-08-1), and genipin (6902-77-8) were purchased from Sigma-Aldrich. The latter stereoisomer was reported to be 1*S*,2*R*,6*S* (PubChem SID 329799931, Fig. 1). The buffer of choice was PBS (cat. number P4417), dissolved from tablets in DEPC milliQ water at a final concentration of 10 mM phosphate buffer, 137 mM sodium chloride, and 2.7 mM potassium chloride (from now on, PBS 1×). RNase A from bovine pancreas (EN0531) was purchased from Thermo Fisher. Ninhydrin (485-47-02, amino acid detection grade) was purchased from Sigma-Aldrich and TNBS (2,4,6-trinitrobenzene sulfonic acid) came from Pierce protease assay (Thermoscientific, 23 263). All disposables were certified pyrogenase, DNase and RNase free, and glassware was autoclaved before use. Sephadex G25 PD10 columns were purchased from GE Healthcare, UK, and each column was used up to ten times and regenerated with a 20× excess of elution volume after every purification round, and kept under sterile conditions. Anti-TdTomato siRNA was purchased from IDT Genomics from a Custom Silencer™ ThermoFisher design. For agarose gel assays, agarose low-melting-point (Promega) and home-made TRIS-acetic acid-EDTA buffer were used (all reagents were purchased from Sigma-Aldrich).

### 5.2 Preparation of genipin-based polymers

Different concentrations (from 1 to 10 mM) of polyamines and glycine (from 1 to 10 molar ratio with genipin) were used to prepare different formulations of the polymer. Briefly, glycine and polyamines were first mixed in 300 μl of 1× PBS, pH 7.4, and finally genipin was added. The reaction was carried on in an excess of oxygen, in 24-well plates, under continuous shaking for a minimum of 20 hours to a maximum of 48 hours, at 25 °C. The reaction time was finally standardized to 36 hours. To avoid excessive evaporation of the solution, neighbouring wells were filled with water. The whole reaction was carried on in a sterile environment. To remove excess polyamines and glycine, the final suspension was eluted, 1 ml at a time, with water with a Sephadex PD10 G50 column, the nanoparticles eluting at fraction 6 and 7. The suspension was subsequently freeze-dried in a Martin Christ freeze drier and powders were rehydrated at 20 mg ml^−1^ in PBS 1× and energetically vortexed after rehydration to allow complete solubilization of the clumps. To avoid spontaneous oxidation of the spermine and spermidine moieties, final suspensions were stored under an argon atmosphere at 4°. According to experimental necessities, the procedure above was upscaled or downscaled. Polymers were named G*x*S5, whereas *x* is the molar ratio of glycine-to-genipin, *i.e.*, 1, 5 and 10.

### 5.3 Quantification of free amine groups

For quantification of free amine groups in G*x*S5 samples, ninhydrin assay was used. Powdered ninhydrin (Sigma-Aldrich) was resuspended at a final concentration of 2 mg ml^−1^ in absolute ethanol, and added in a 1 : 5 volume/ratio with the sample, to sample/standard containing tubes. Tubes were heated up at 85° for five minutes and allowed to cool at room temperature for one hour. The final absorbance was recorded at 290, 350, 400 and 570 nm. Spermine from a 0.50 M water stock was used as the standard for calibration curve determination, within a range of 0.08–15 mM. Each sample was prepared in triplicate and normalized for intrinsic polymer absorbance at the corresponding wavelength. The nitrogen amount was defined as the quantity of ninhydrin-reacted amine groups in polymers, quantified at 350 and 570 nm. For TNBS (2,4,6-trinitrobenzene sulfonic acid), a stock in methanol (5% w/v) was stored at 4°, and diluted up to 0.1 w/v % for the assay procedure. All experiments for the TNBS assay were performed in 0.1 M sodium bicarbonate as the working buffer, pH 8.5. Glycine, from a water stock of 0.50 M, was used as the standard to build a calibration curve. Briefly, the solution and glycine standard were diluted in 1.5 ml Eppendorf tubes in a working buffer up to 0.25 ml of volume, and 0.125 ml of 0.1% TNBS was added. Tubes were incubated at 37° for two hours, until the development of a strong orange colour. Absorbance was recorded at 335 nm, measuring therefore picric acid formation. All samples were run in triplicate. The conventional use of sodium dodecyl sulphate was avoided to maximize the sensitivity.^53^

### 5.4 Complexation of siRNA–G*x*S5

TdTomato siRNA was mixed at various nitrogen-to-siRNA phosphate (N/P) molar ratios with G*x*S5 polymers, vortexed for 10 seconds, and incubated for at least half an hour at room temperature, in sterile-filtered, autoclaved DEPC-PBS 1× (pH 7.4). Samples were used without further purification. For the sake of clarity, where complexed with siRNA, G*x*S5 are referred to as “polyplexes”.

### 5.5 Dynamic light scattering and *ζ*-potential measurements

Particle size, polydispersity and *ζ*-potential measurements were all carried out in an Anton Paar Litesizer within 1 week from the rehydration of samples. A total of 1000–650 μl was added in an Omega Cuvette and repeated measurements (*n* > 5) were carried on to ensure statistical repeatability. A number-weighted, built-in algorithm was used for all particle size measurements.

### 5.6 Determination of P580 molar absorptivity

For the determination of the molar absorptivity of the product forming at 580 nm (P580) different concentrations of genipin (from 0.168 mM to 0.5 mM) were allowed to react with 5 mM spermine and the final OD at 580 nm was plotted against the initial genipin concentration and the absorbance was recorded, on a Greiner Bio-One UV-Star™ 96-well Microplates, on a Synergy Microplate (Biotek) reader. After 36 hours of reaction, final absorbance values at 580 nm and 375 nm were used to build a calibration curve, whereas the slope represents the molar absorptivity *ε* of P580 and at P375. The following pathlength-correction protocol was applied in order to correct the absorbance for the volume present in the microwell and standardize all the OD values to a virtual 1 cm quartz cuvette.14
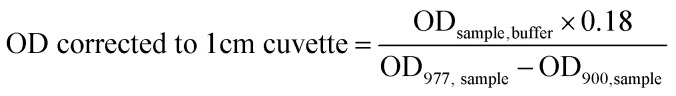


### 5.7 Agarose gel assays

To assess the stability of the complexation of the polymers with TdTomato siRNA, complexes at different P/N ratios were loaded in a 2.5% agarose gel, pre-stained with Midori Green (Nippon Genetics), and allowed to run for 20 minutes at 70 V in TAE buffer 1× (pH 8.5, TRIS 40 mM, acetic acid 20 mM, EDTA sodium salt 1 mM). Briefly, 10 μl, in PBS, contained 1 μM of siRNA, which was kept constant for each P/N polyplex, while the amount of polymers was varied accordingly. The whole volume for each P/N was then loaded in the agarose gel. Subsequently, bands were visualized in a ChemiDoc gel imager. Heparin sodium salt was used to de-complex the siRNA from polymers at a final concentration of 5 mg ml^−1^. A similar procedure was followed after incubation of polymers at 1 : 25 and 1 : 50 P/N ratios with RNAse A for 4 and 24 hours. The DNA ruler is 10 bp DNA ladder (Thermo Fisher Scientific, Waltham, MA, USA; cat. 10 821–015).

### 5.8 TEM imaging conditions and image analysis

An unfixed sample was dropcast onto a copper-nickel 200-mesh TEM grid (Agar Scientific, AGS138) and characterized using a transmission electron microscope (TEM Jeol JEM-2010F), operated at 200 k eV. For image analysis, ImageJ software was used on a *n* > 10 micrographs.

### 5.9 Potentiometric pH titration

Freeze-dried suspensions of polymers were rehydrated with water at a final concentration of 5 mg ml^−1^. Subsequently, the pH was adjusted at around pH 2 with 0.5 M HCl and suspensions were back-titrated with 0.1 M NaOH to pH 10. Final back-titration profiles were normalized for the dilution of the polymer.

### 5.10 Spontaneous release assay

To assay the eventual spontaneous de-complexation of siRNA from G*x*S5 polymers, a Quantifluor dye-based assay was established. A total of 0.1 μM of siRNA was allowed to complex with RNA Quantifluor dye (from RNA Quantifluor kit, Promega E3310) in PBS 7.4 for 20 minutes, and baseline fluorescence was recorded. Subsequently, an amount corresponding to a final 1 : 2, 1 : 25 and 1 : 50 phosphate/nitrogen ratio amounts of G1S5, G5S5, and G10S5 was added to siRNA, and incubated for an additional 20 minutes before recording the fluorescence. To assess the de-complexation under conditions mimicking the endosomal environment, 1 M HCl was added to the wells dropwise until pH 5 was reached, carefully measuring the pH with a microprobe. The evolution of fluorescence was recorded during 1 hour of incubation and fluorescence values were normalized for a free-siRNA control for pH-related hypochromic shifts. To assess the complete release of siRNA from nanoplexes, 5 μg ml^−1^ and 2 mg ml^−1^ of heparin (from porcine pancreas, Sigma-Aldrich) were added to the samples and consequent fluorescence spikes were recorded. The final de-complexation is expressed as the increase of fluorescence occurring upon freeing of siRNA by the change in pH or heparin addition.

### 5.11 Characterization of FT-IR polymers

Roughly 2 mg of G*x*S5 polymers were freeze-dried in the absence of salts and argon-flushed before analysis. A GX Fourier-transform IR spectrometer (PerkinElmer) equipped with a Model 300 photoacoustic detector (MTEC) was used and spectra were transformed in absorbance units and normalized for the strong absorbance peak at 542 cm^−1^. Spectra were recorded from 450 cm^−1^ to 4000 cm^−1^.

### 5.12 Cell culture conditions and cytotoxicity evaluation

Cell lines B16F10 (CRL-6475) 293T (CRL-3216), CT26 (CRL-2638), and 4T1 (CRL-2539) were originally purchased from ATCC (VA, US) and the B16F10-tdTomato cells were a kind gift from Dr Muriel Golzio (The Institute of Pharmacology and Structural Biology, IPBS-Toulouse). L929 cells were originally purchased from Merck (NJ, USA). B16F10, 293T, B16F10-tdTomato and L929 cells were cultured in Advanced Dulbecco's modified MEM medium (DMEM, Gibco, Thermo Fisher Scientific, VA, US). CT26 and 4T1 cells were cultured in Advanced Roswell Park Memorial Institute 1640 medium (RPMI-1640, Gibco, Thermo Fisher Scientific, VA, US). All media were supplemented with GlutaMAX (100×, Gibco), 5% foetal bovine serum (FBS, Gibco), and penicillin–streptomycin (100×, Sigma-Aldrich, Merck, Darmstadt, Germany). The cells were routinely tested for mycoplasma infection by MycoAlert™ PLUS Mycoplasma Detection Kit (Lonza, Basel, Switzerland) and were mycoplasma free. Cells (1500/well) were seeded in a 96 well plate (VWR, PA, US), allowed to grow overnight, and later incubated for 48 hours with increasing concentrations of polymers. Next, 10 μl per well of Presto Blue reagent (A13261) was added to cells and incubated at 37° for 1 hour. Finally, the emitted fluorescence was read with a Cytation 1 system (BioTek Instruments), VT, USA. HaCaT human keratinocyte cells (ATCC PCS-200-011) were confluently grown from a frozen low passage in a T25 flask with 10 ml of full medium containing DMEM supplemented with 10% FBS and 1% penicillin streptomycin. The diluted HaCaT cells were seeded (at 20 000 cells per well) onto a tissue-culture treated 24-well plate until 80% confluency. At this point 10 vol% of Presto Blue Cell Viability (fluorescence/emission at 590/560 nm measured after 1 hour of incubation) was used to measure cellular growth before treatment. After removing the dye (2× washing with PBS) a fresh full DMEM medium with different concentrations of tested polymers were added to the cells. Cells were incubated with polymers (humidified 5% CO_2_ atmosphere at 37 °C (in MCO-19AIC(UV)-PE incubator, Panasonic)) for 3 days. After that, DMEM medium with the tested polymer was removed (2× washed in DPBS), replaced with fresh full DMEM medium and cell proliferation was further monitored for the next three days. Presto Blue Cell Viability Reagent was used to detect viability of cells after 3 days incubation with polymers as well as during the following 3 days incubation without the polymer. The test was performed at least in two independent experiments, each time using three parallels for each sample. References were tested compounds in supplemented DMEM without cells, pure dye in DMEM, cells without tested compounds (as a negative control), and cells with Triton 100 (as a positive control).

### 5.13 Hemolysis test

Red blood cells were obtained from the Transfusion Center of Ljubljana (Ethical approval number 0120-592/2020/7), and belonged to two young anonymous males, A RhD+ blood type, supplemented with CPD-SAGM preservative, 70% v/v hematocrit, accounting for 4 × 10^9^ RBCs per ml. Plasma from whole blood purification was also obtained and used for fluorescence studies. The cells were used within three days, at final 5% haematocrit, and incubated with 0.0035–0.02 mg ml^−1^ of G*x*S5 polymers for 4 hours at 37%, *n* = 3 for each concentration. Red blood cells incubated only with PBS were used as the negative control. After incubation, cells were pelleted and the absorbance of the surnatant was measured at 541 nm.

### 5.14 Flow cytometry

To ascertain any differences in B16F10 cells' uptake among siRNA-complexed and empty polymers their internalization was determined with flow cytometry using a BD FACSymphony™ A3 Cell Analyzer. The cells were incubated for 48 hours with 1 : 2, 1 : 25 and 1 : 50 P/N G*x*S5 polymers and the corresponding concentration of uncomplexed polymers. After 48 hours, cells were trypsinized and resuspended in phenol-free-DMEM, incubated with 3 μg ml^−1^ of DAPI to exclude dead cells. From the live cell population the percentage of fluorescent positive cells, indicating the uptake of polymers, was determined. Additionally, the median fluorescence intensity (MFI) in the PE channel of the live-cell population was determined. Similarly, the silencing efficacy of polyplexes was determined with flow cytometry. Briefly, 50 000 cells were seeded on a 24 well plate, and let to grow overnight. Transfection was carried out with a total amount of 10 picomoles of siRNA per well. Free polymers, with the same final concentration, were used as the negative control. After 72 h hours of growth, the cells were trypsinized, washed in 1× PBS and stained with DAPI to label dead cells. From the live cell population, the percentage of tdTomato positive cells and their MFI was determined. The MFI of treated groups was then normalized to the negative control group.

### 5.15 Determination of silencing efficacy with q-RT PCR

At 1 : 2, 1 : 25 and 1 : 50 siRNA backbone phosphate/total nitrogen content ratios, polyplexes were used to transfect B16F10 murine melanoma cells, expressing tdTomato fluorescent protein. Before transfection, polyplexes were mixed with the same volume of OPTImem medium. Polyplexes were dropped in each well without mixing. RNAiMAX, a state of the art polymer for lipofection, was used as the positive control. Briefly, 50 000 cells were seeded on a 24-well plate, and let to grow overnight. Transfection was carried out with a total amount of 30 picomoles of siRNA per well, on *n* = 3 biological replicates. Free polymers, with the same final concentration, were used as the reference for tdTomato and β-actin gene fluctuations. After 48 hours of incubation, total RNA was extracted with a VWR pGold Total RNA kit and quantified on a Take3 plate on a Cytation 1 (BioTek Instruments, VT, USA). A Scriptase II kit (Nippon Genetics, LS53) was used to synthesize cDNA at a final concentration of 50 ng μl^−1^. Finally, a Power Sybr Green master mix (Applied Biosystem, 4 367 659) was used for qPCR on a Quantstudio 3 apparatus and the 2ΔΔ*C*_T_ method was used for relative quantification (Table 5 for primer sequence). *N* = 6 technical replicates were used for *C*_T_ quantification.

**Table 5:** Primer sequence used for quantification of knockdown

Primer	Sequence
TdTomato – forward	5′-GACAACAACATGGCCGTC-3′
TdTomato – reverse	5′-GGTCTGGGTGCCCTCGTA-3′
β-actin – forward	5′-GAAGTGTGACGTTGACATCC-3′
β-actin – reverse	5′-ACTCATCGTACTCCTGCTTG-3′

### 5.16 Statistical analysis

All statistical analysis were run in Graphpad 10. For tdTomato and β-actin gene expression, CT data were trimmed with a built-in Graphpad 10 outlier recognition algorithm. All datasets were tested for normality or lognormality before any stat test. ANOVA, two or one way, was used for normally distributed data, while non-parametric Kruskall–Wallis was used for lognormal. ANOVA-One Way was used to assess the statistical significance of the data on the log10 of fold changes against the control (*n* ≥ 6). For all purposes, significance was determined as *p* ≤ 0.05*, *p* ≤ 0.01**, *p* ≤ 0.001***, and *p* ≤ 0.0001****.

## Ethical approval

For the hemolysis test, human red blood cells were used, and obtained from the Transfusion Center of Ljubljana, as per Ethical approval number 0120-592/2020/7 (National Medical Ethics Committee of Slovenian Republic).

## Consent for publication

All authors have read the manuscript and expressed their consent for publication.

## Author contributions

All authors approved the submitted version. G. D. P. made the major contribution in conceptualization, experimental part, data analysis, figure preparation and writing of the manuscript. T. B. and B. M. provided help in cell culture and manuscript revision. M. V. contributed to Fig. 7 and ESI Fig. S12.† G. S. contributed during revision and funding acquisition. N. K. provided all TEM pictures and played a major role in mentoring, conceptualization, revision and funding acquisition.

## Conflicts of interest

The authors declare that they have no competing interests.

## Supplementary Material

NA-006-D4NA00363B-s001

NA-006-D4NA00363B-s002
