# Supplementary material for: Efficient siRNA delivery to murine melanoma cells via a novel genipin-based nano-polymer
Source: Nanoscale Adv. 2024 Jul 19;6(18):4704–23. doi: 10.1039/d4na00363b (PMC11386170; doi:10.1039/d4na00363b)
Supplement: NA-006-D4NA00363B-s001 [file NA-006-D4NA00363B-s001.pdf]

Electronic Supplementary Material (ESI) for Nanoscale Advances.
This journal is © The Royal Society of Chemistry 2024

Supplementary materials

Efficient siRNA delivery to murine melanoma cells via a novel genipin -based nano-polymer

Giulia Della Pelle [1,2], Tim Bozic [3], Marija Vukomanović [4], Gregor Sersa [3, 5], Bostjan Markelc [3], Nina Kostevšek [1,2]

[1] Department for Nanostructured Materials, Jožef Stefan Institute, 1000 Ljubljana, Slovenia
[2] Jožef Stefan International Postgraduate School, 1000 Ljubljana, Slovenia
[3] Department of Experimental Oncology, Institute of Oncology Ljubljana, 1000 Ljubljana, Slovenia
[4] Advanced Materials Department, Jožef Stefan Institute, 1000 Ljubljana, Slovenia
[5] Faculty of Health Sciences, University of Ljubljana, Zdravstvena pot 5, SI-1000 Ljubljana, Slovenia

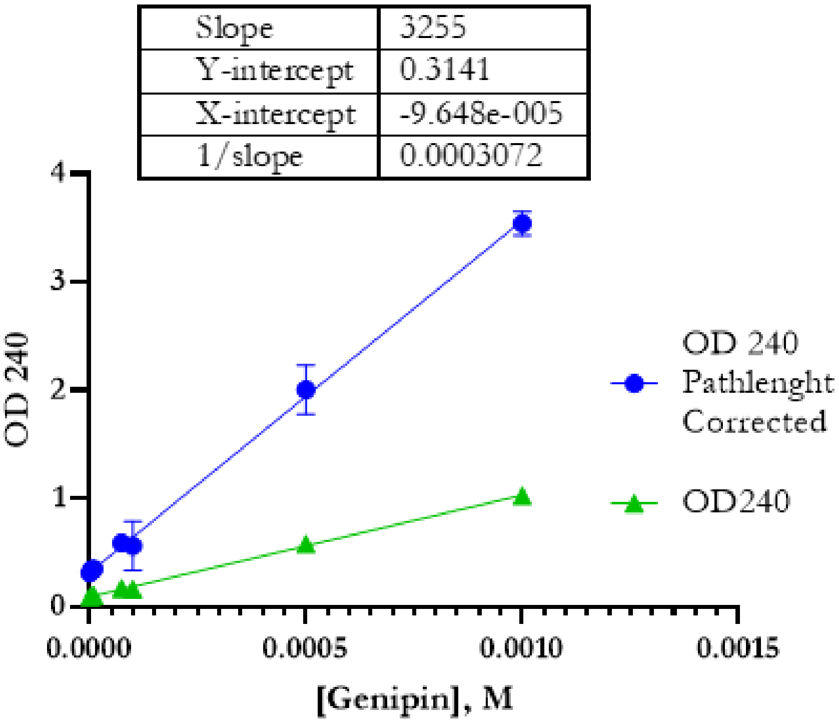

Figure S1: Calibration curve of genipin in 300 µl as in "Kinetic evaluation of genipin reaction with two naturally occurring polyamines" and corrected for a virtual 1-cm pathlength cuvette with equation (14).

[Genipin], M	υ (M/h), 240 nm	a_1
0.000168	-0.0000557	1.57 ± 0.25

0.000252	-0.000133
0.000335	-0.000309
0.000504	-0.000546

Table S1. Initial reaction speed monitored at 240 nm versus different initial genipin concentrations, i.e., spermine was the isolated reactant. Reaction order a_1 was derived by equation (3) and averaged for all reactions. Spermine concentration is 0.00083 M in all cases.

**Primary amine groups donor**	**[R-NH2], M**	**υ (M/h), 240 nm**	**b_1**
**Spermine**	0.000166667	9.197E-07	1.16 ± 0.32
**Spermidine**	0.000166667	0.000003554	
**Mix 1:1 m/m**	0.000166667	0.000009269	
**Spermine**	0.000833333	0.000004941	
**Spermidine**	0.000833333	0.0000154	
**Mix 1:1 m/m**	0.000833333	0.00002301	
**Spermine**	0.001666667	0.00001137	
**Spermidine**	0.001666667	0.00002944	
**Mix 1:1 m/m**	0.001666667	0.00004428	

Table S2. Initial reaction speed monitored at 240 nm versus different amine donor initial concentrations, i.e. genipin was the isolated reactant, with a concentration of 0.00083 M. Amine groups sources are in brackets. Reaction order b_1 was derived by equation (4) and averaged for all reactions.

**[Genipin], M**	**υ (M/h), 580 nm**	**a_2**
0.000168	0.03176	1.51 ± 0.8
0.000252	0.05277	
0.000336	0.1003	
0.000504	0,3216	

Table S3. Initial reaction speed monitored at 580 nm versus different genipin initial concentrations, i.e. spermine being the isolated reactant. Reaction order a_2 was derived by equation (3) and averaged for all reactions.

Primary amine groups donor	[R-NH$_2$], M	υ (M/h), 580 nm	b $_2$
**Spermine**	0.000166667	0.000662	0.53 ± 0.17
**Spermidine**	0.000166667	0.000818	
**Mix 1:1 m/m**	0.000166667	0.001391	
**Spermine**	0.000833333	0.001278	
**Spermidine**	0.000833333	0.001669	
**Mix 1:1 m/m**	0.000833333	0.002188	
**Spermine**	0.001666667	0.001307	
**Spermidine**	0.001666667	0.001052	
**Mix 1:1 m/m**	0.001666667	0.00274	

Table S4. Initial reaction speed monitored at 580 nm versus different amine donor initial concentrations, i.e., genipin being the isolated reactant. Amine groups sources are in brackets. Reaction order a_2 was derived by equation (4) and averaged for all reactions.

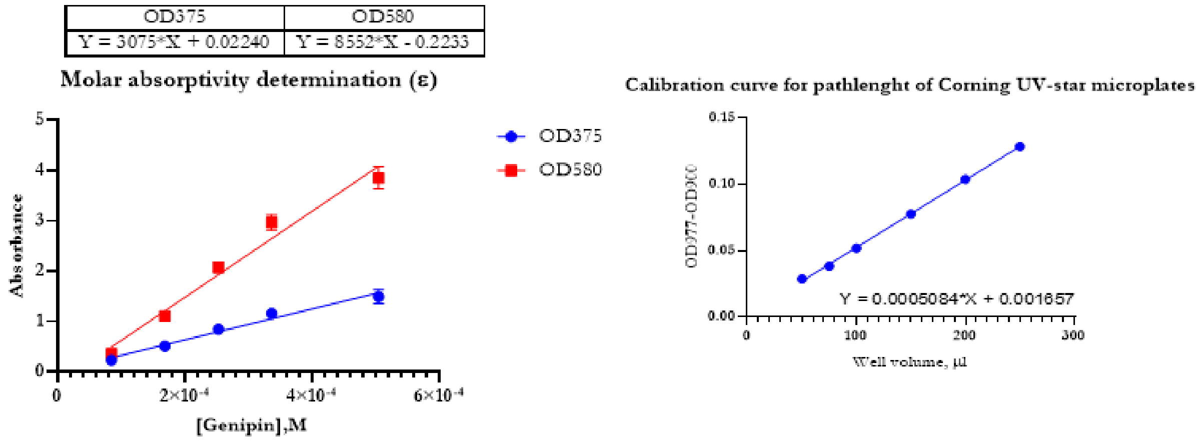

Figure S2: a) Plot genipin vs plateau OD375 nm and OD580 value. ε is represented by the slope, as for Lambert and Beer law; b) Calibration curve for pathlength correction used to determine molar absorptivity of GxS5 products.

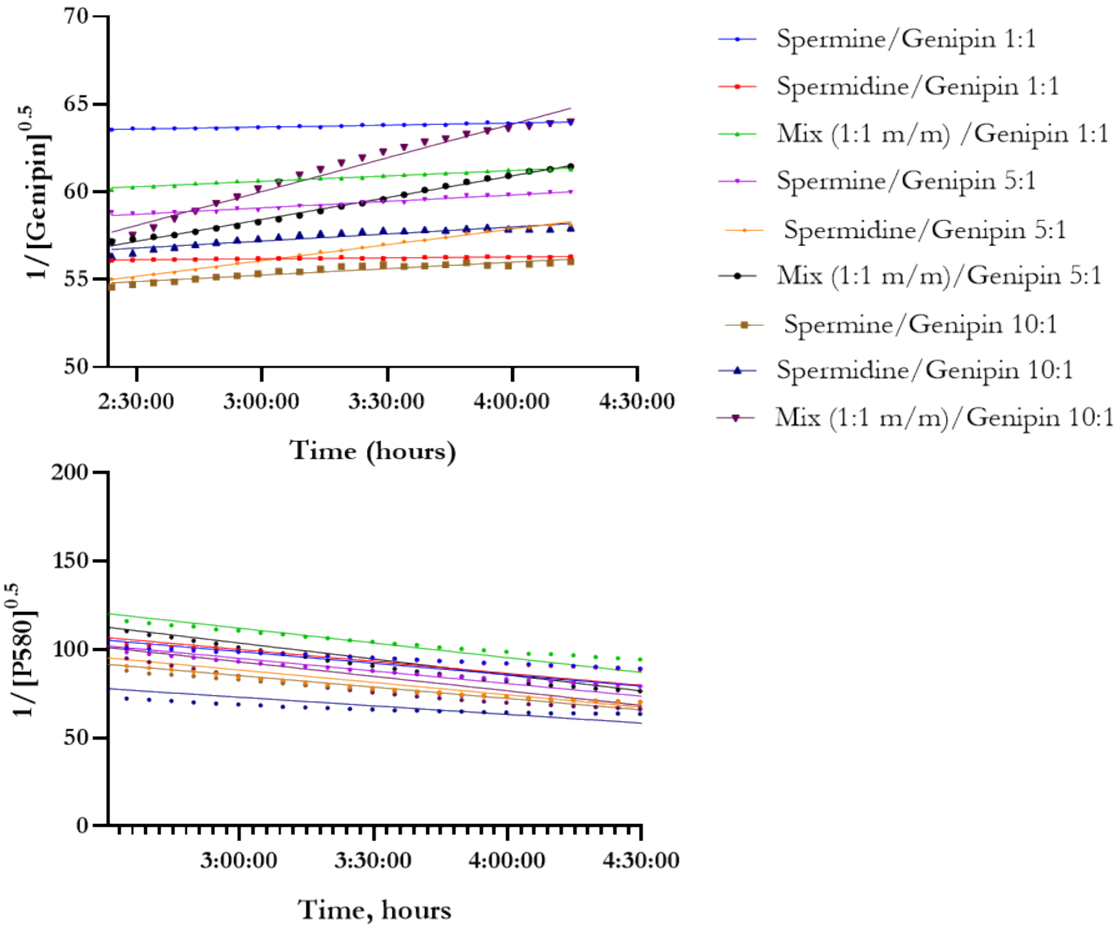

Figure S3. Above, graph for genipin consumption monitored at 240 nm corresponding to a reaction order of 3/2, reworked according to [1] as of equation (8). In legend, amine groups source and molar ratio with genipin. Below, graph for P580 formation, corresponding to experimental reaction order ½. Slope is k.obs/2, as of equation (9). Colours refer to the same sample for both graphs.

Molar ratio with Genipin	ζ potential, mV
**Spermine, 1:1**	14.78 ± 3.18
**Spermidine, 1:1**	3.4 ± 1.23
**Mix 1:1, 1:1**	10.67 ± 2.33
**Spermidine, 1:5**	5.68 ± 1.59
**Mix 1:1, 1:5**	7.68 ± 1.68
**Spermine, 1:5**	18.96 ± 0.95

**Spermidine, 1:10**	12.46 ± 2.05
**Mix 1:1, 1:10**	18.94 ± 0.65

Table S5. ζ potentials of polymeric suspensions resulting from reaction of genipin with multiple combinations of spermine and spermidine.

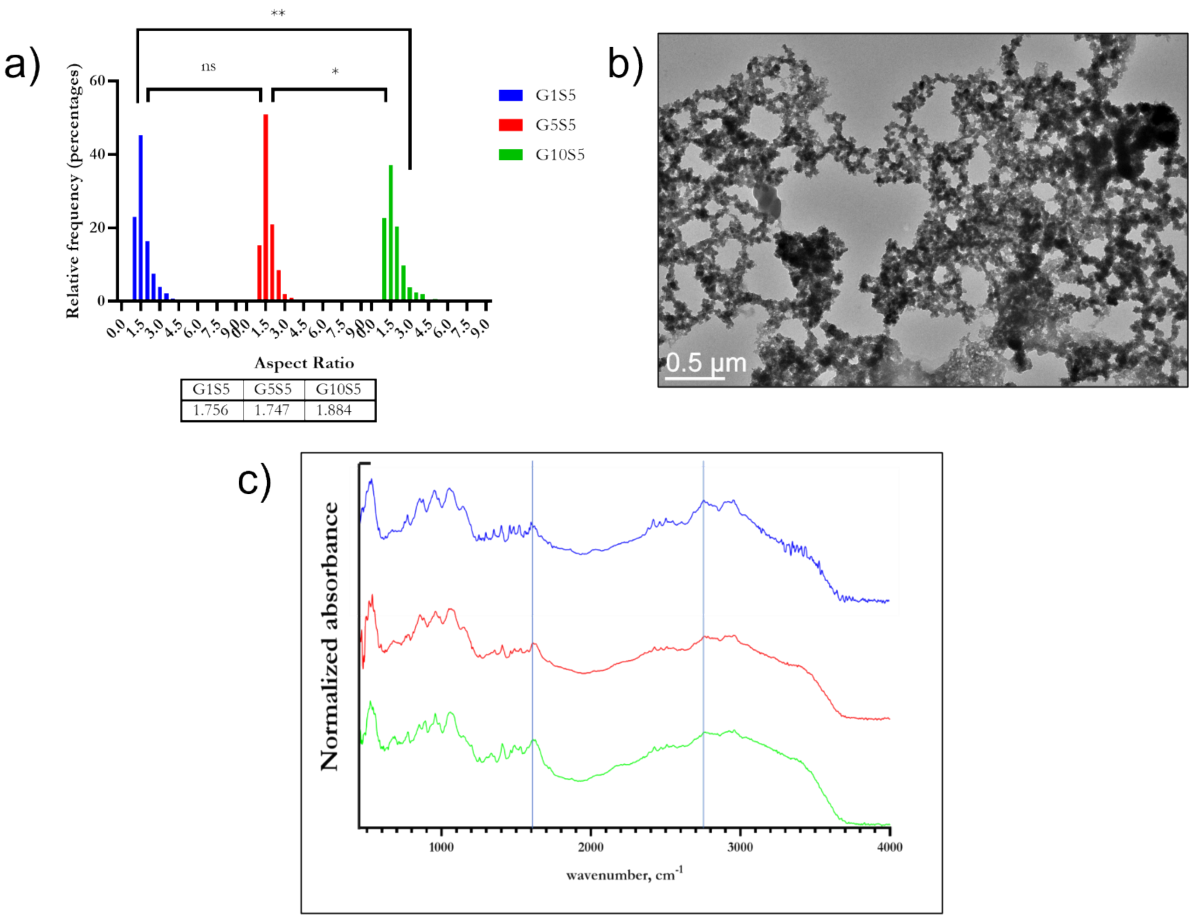

Figure S4 a)Aspect ratio frequency distribution of GXS5 nanoparticles from TEM micrographies. The datasets included 10 micrographies at 6000x magnification for each polymer while measured nanoparticles were, 1213, 1149 and 1187 respectively for G1S5, G5S5, G10S5. Aspect ratio was derived from ImageJ software built-in functionality. The harmonic mean of aspect ratios is reported below the plot. b) TEM micrograph of G0S5 polymer. No monodisperse, well-defined particle suspension was detectable.c)FT-IR spectra of, from top to bottom, G1S5, G5S5 and G10S5.

Fourier-Transform Infrared (FT-IR) Spectroscopy

The Fourier-transform infrared spectroscopy (FTIR) data are presented in Figure S4c. The spectra are consistent with those already present in the literature for genipin/spermine amine crosslinking [2,3]. Although the spectra of GxS5 did not differ dramatically, a few differences should be highlighted. The peaks corresponding to primary and aliphatic amines (3500–3100 cm^{-1}) are much more pronounced in G1S5, but minimal in G10S5. The intramolecular C–H stretch signal at 2700 cm^{-1} appears to be stronger for the lower glycine molar fraction. In all three samples, the alcoholic function stretching of the genipin monomer is present as a small hump at 3500 cm^{-1}, while the O–H bending peaks around 1345 cm^{-1} are equally intense. At

shorter wavenumbers, the carboxylic group stretching at 1700 cm^{-1}, on the contrary, appeared to be stronger for a higher glycine molar fraction. A peak corresponding to lactone heterocycle, belonging to the amide moiety of reacted iridoid ring of genipin, is present at 1645 cm^{-1} in all three samples [3]. Regions between 1600 cm^{-1} and 500 cm^{-1} are challenging to interpret. Whereas the N-H amine bending signal at 1615 cm^{-1} increases from G10S5 to G1S5, C–O stretching, carboxylic acid C–H bending, and C–N cyclic stretching are heavily superimposed in the 1300 cm^{-1} and 1200 cm^{-1} region. However, the carboxylic C–H at 1415 cm^{-1} appears neater in the G10S5 due to presence of a glycine-carboxylic-free moiety. Lastly, no appreciable differences are evident in the C=C and C–H bending region from 1100 cm^{-1} onwards. Due to the overlapping signals in the range 1250–1300 cm^{-1}, it was not possible to distinguish those arising only from tertiary amines, i.e., amines derived from the nucleophilic attack of genipin on spermine, the substitution of the oxygen at position 2 and the final crosslinking with another genipin moiety. Therefore, despite some subtle differences among the three spectra, FT-IR does not have sufficient resolution to investigate our polymers' fine structure thoroughly, and further analysis will be needed.

1 S. K. Upadhyay, *Chem. Kinet. React. Dyn.*, 2006, 1–256.

2 M. F. Butler, Y. F. Ng and P. D. A. Pudney, *J. Polym. Sci. Part A Polym. Chem.*, 2003, **41**, 3941–3953.

3 F.-L. Mi, H.-W. Sung and S.-S. Shyu, *J. Polym. Sci. Part A Polym. Chem.*, 2000, **38**, 2804–2814.

Quantification of mass concentration of GxS5 polymers using OD at 580 nm

After assessing the repeatability of our synthesis and purification protocol, we aimed to establish a correlation between the molar concentration of P580 and the weight. After elution in water with Sephadex G50 PD10 columns (Materials and Methods), polymers were freeze-dried for a maximum of 48 hours and subsequently rehydrated in PBS 1× at 20 mg/ml. With a series of serial dilutions, absorbance at 580 nm was recorded. A linear relation for molar concentration of P580 vs weight concentration was generated (Figure 5Sa) and used for all P580 quantifications in subsequent experiments. [P580] (Figure 2s) was obtained from absorbance using Lambert & Beer relation, being ε = 8.5 103 mol^{-1} l^{-1}, similar to the reported molar absorptivity of genipin reacted with lysine [23], and it is used for all the further calculations whereas molar concentration was needed.

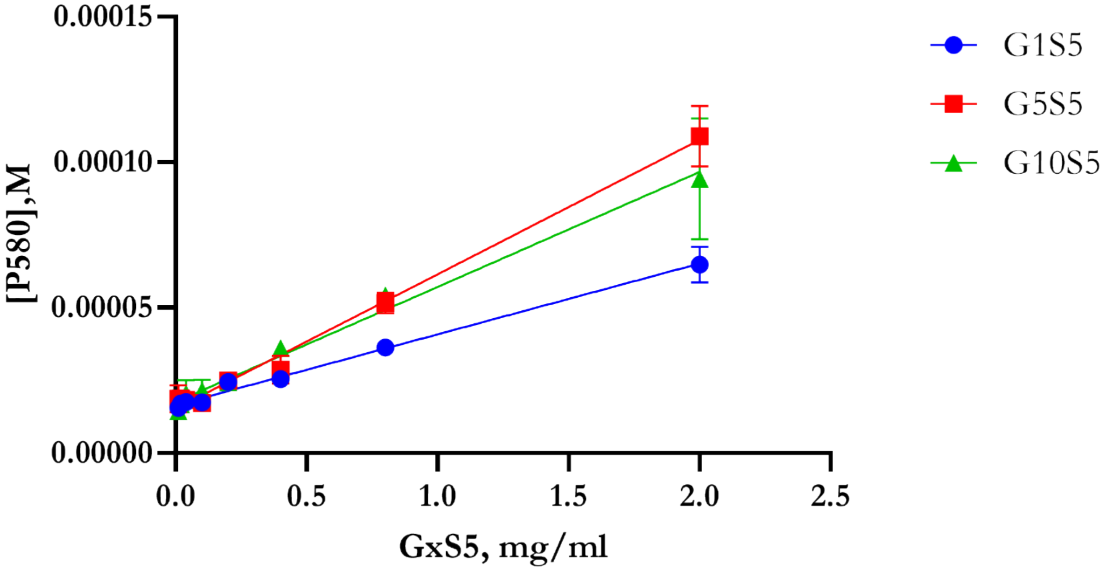

G1S5	G5S5	G10S5
Y = 2.436e-005*X + 1.647e-005	Y = 4.621e-005*X + 1.526e-005	Y = 3.953e-005*X + 1.759e-005

Figure S5. Relation between weight concentration (in mg/ml) of rehydrated GxS5 polymers, and P580 molar concentration. R2 was found to be, for G1S5, G5S5, G10S5, 0.97, 0.97 and 0.98, respectively.

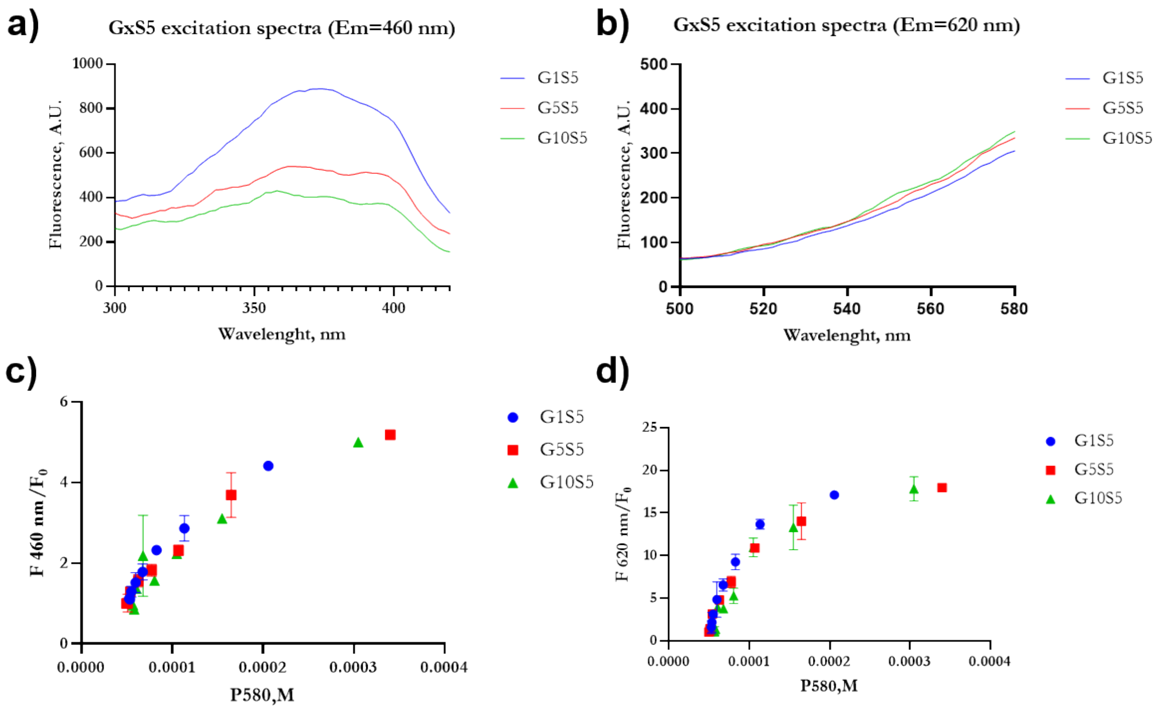

Figure S6. Fluorescence excitation spectra, with fixed emission at 460 nm (a), and 620 nm (b). Fluorescence emission respectively a) at 460 nm ; b) at 620 nm versus [P580],M.

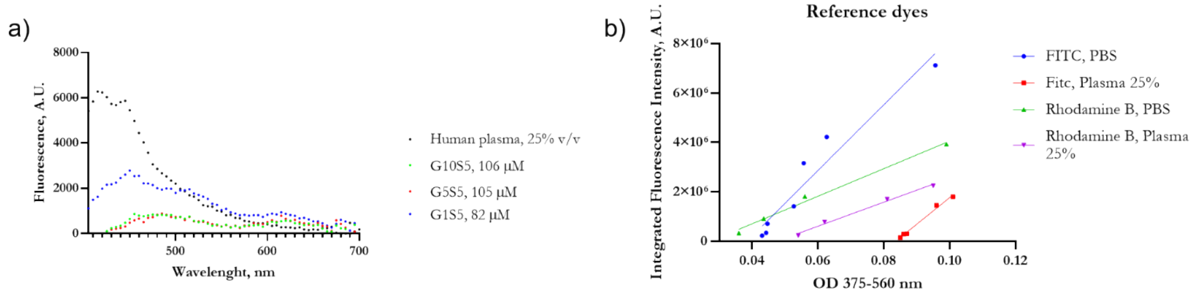

Figure S7. a) Baseline-removed emission spectra of GXS5 (excitation at 375 nm) in 25% v/v of human plasma compared to baseline. b) Slopes of fluorescence reference dyes, FITC and Rhodamine B.

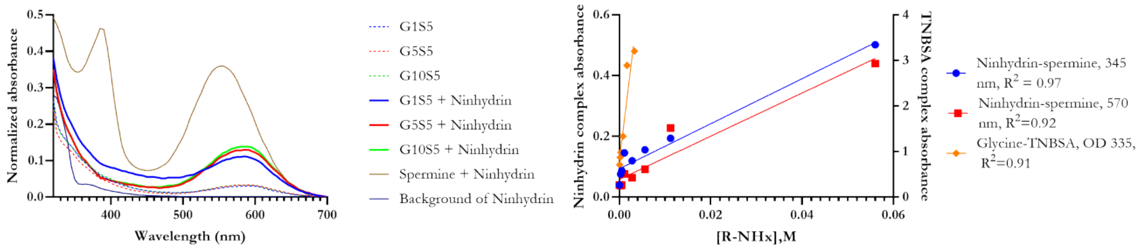

Figure S8. a) Absorbance spectra of polymers alone and complexed with ninhydrin. b) Calibration curves of spermine at 345 nm and 570 nm with ninhydrin, and relative to glycine in presence of TNBS.

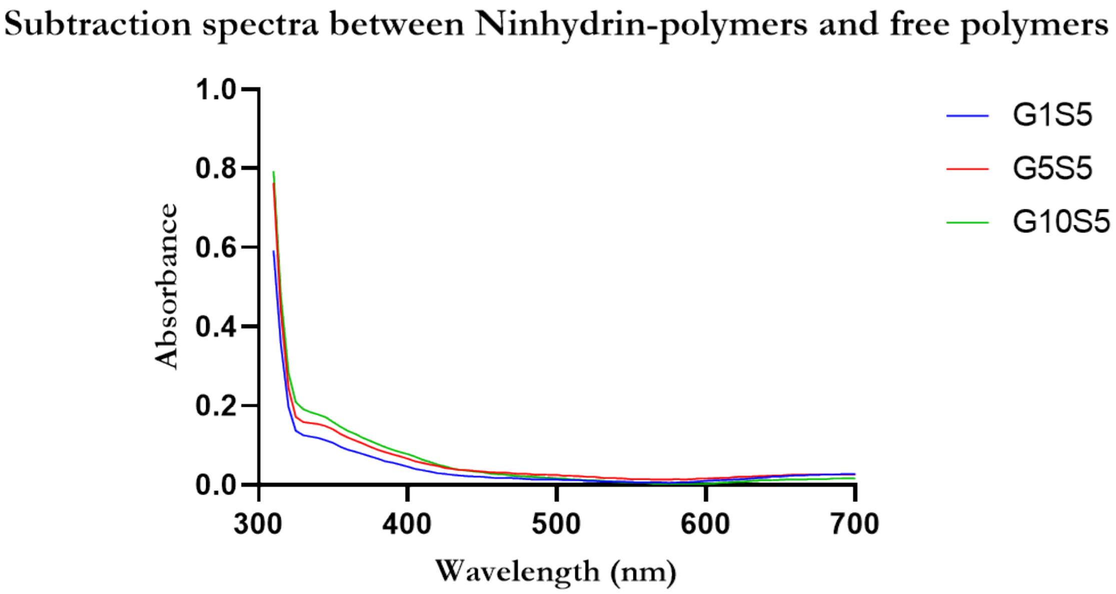

Figure 9S. Subtraction spectra resulting from GxS5 absorbance and their spectra after complexing with ninhydrin. The faint hump at 340–350 nm was used for quantification via spermine-generated standard curve.

	**Fold variation in P1/P2 ratio from pH 7.4 to pH 5**	**Fold size increase in P1**	**Volume increase in P1**
**G1S5**	1,93	1,92	5,64
**G5S5**	1,51	1,95	9,45
**G10S5**	1,15	2,24	11,29

Table S6. Fold variation of relative abundancies of P1 and P2, increase in terms of diameter of particles from pH 7.4 to 5, and relative increase in volume.

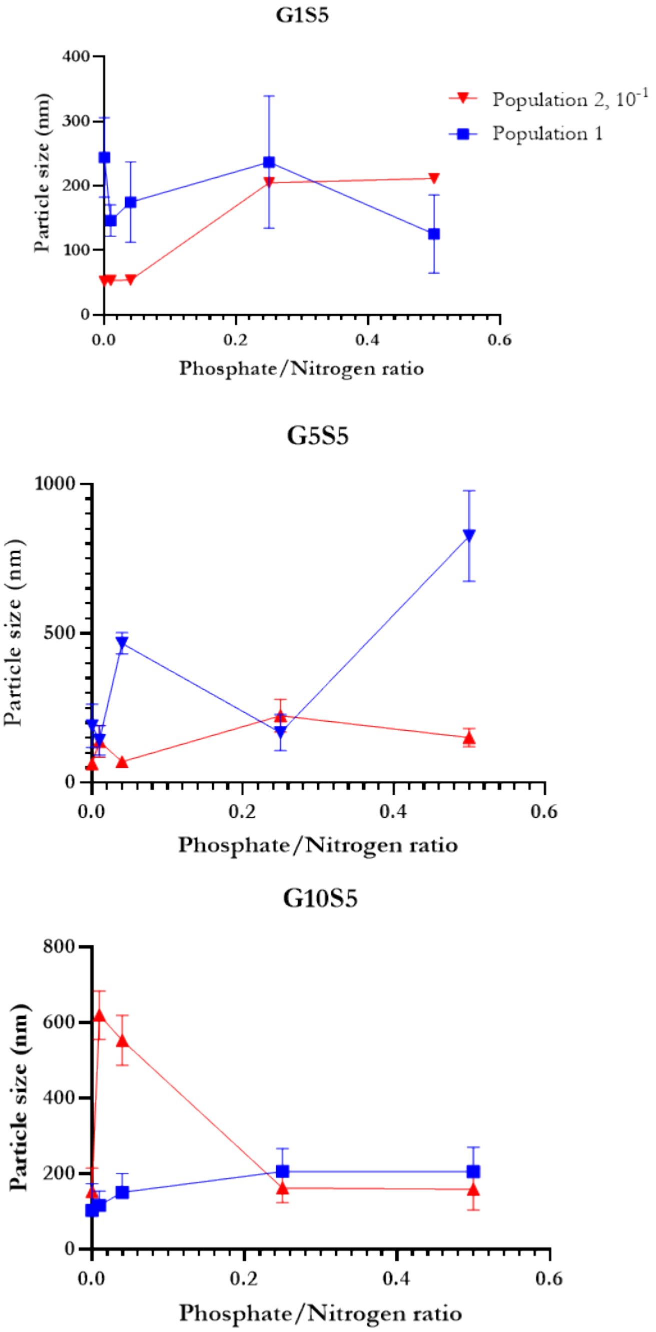

Figure S10. Evolution of particle size of GxS5 polymers, each divided in two different nanoparticle populations, according to P/N ratio.

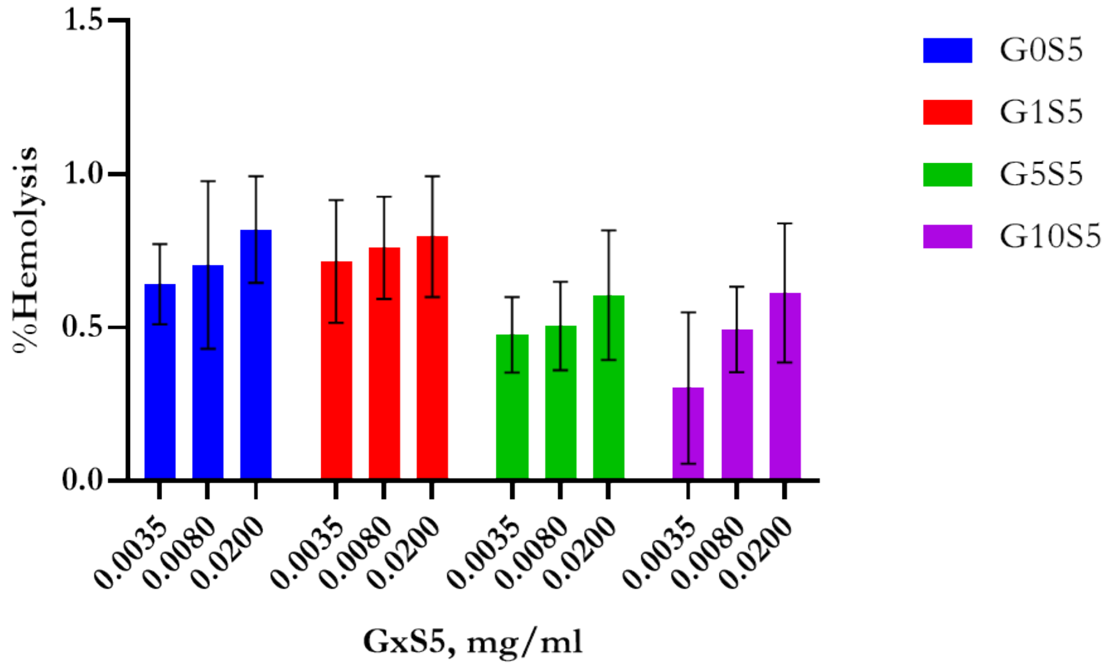

Fig S11.Percentage of hemolysis of GxS5 polymers.

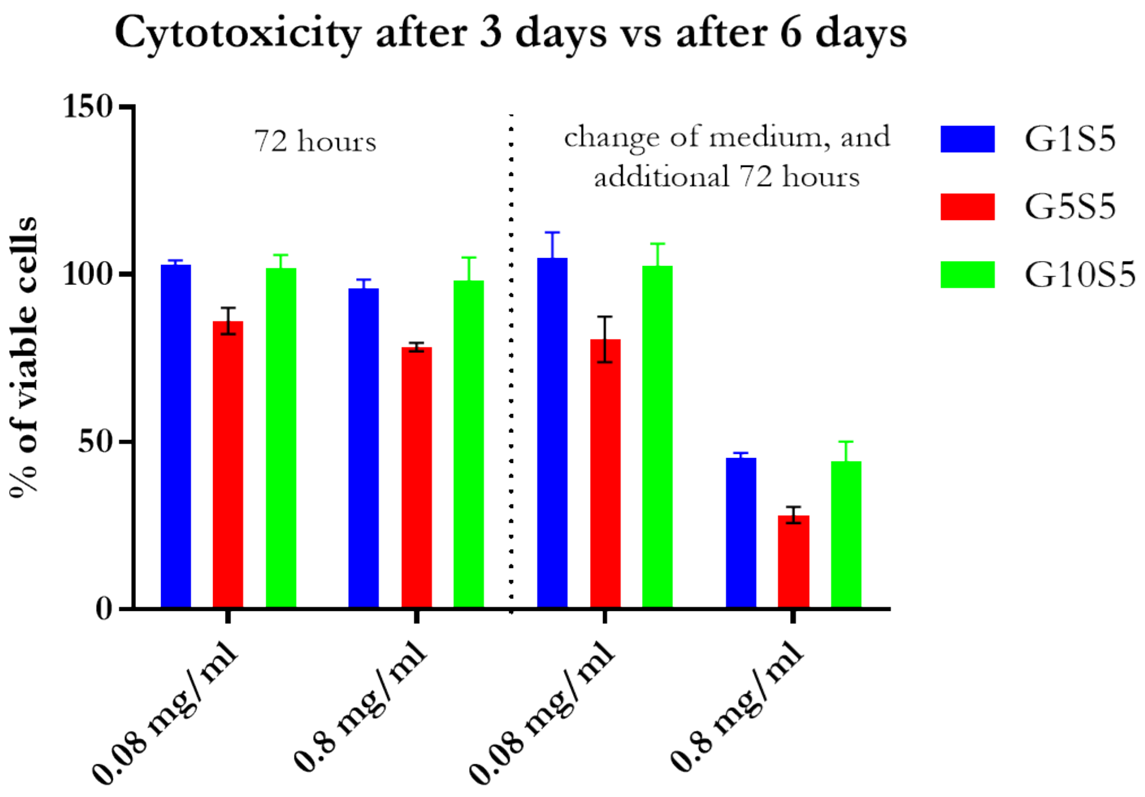

Fig S12. Cytotoxicity on HaCaT cells.

Ratio with siRNA phosphates	Polymer	Concentration in wells (mg/ml)
	G1S5	0.0033
	G5S5	0.0032
1:2 P/N	G10S5	0.0027
	G1S5	0.0357
	G5S5	0.0310
1:25 P/N	G10S5	0.0264
	G1S5	0.0868
	G5S5	0.0774
1:50 P/N	G10S5	0.0682

Table S7. Final concentrations of GxS5 used for B16F10 transfection, in a 24 well-plate, each filled with 1 ml of culture medium.

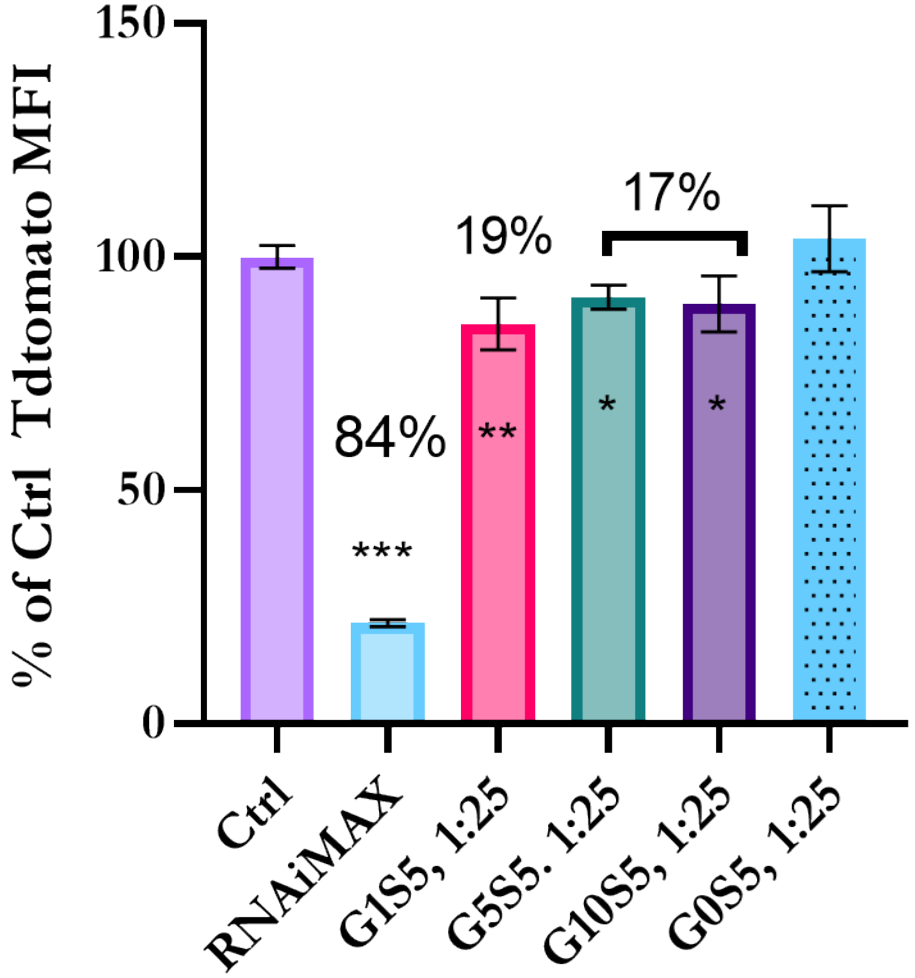

Figure S13. Remaining MFI of tdTomato, normalized for the control, for GxS5 polymers complexed with siRNA at 1:25 P/N. The final siRNA amount used to transfect cells was 10 picomoles.

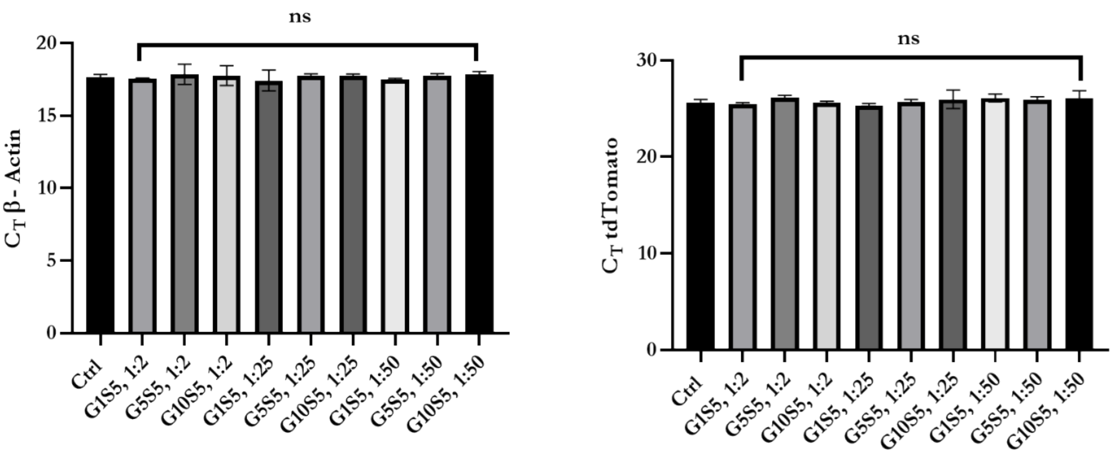

Figure S14. Average β-actin for siRNA/GxS5 complexes and tdTomato C_T at various GXS5 concentrations (as per Table S7) un-complexed with siRNA, used as negative control.
