# Supplementary figures and images for: Efficient siRNA delivery to murine melanoma cells via a novel genipin-based nano-polymer
Source: Nanoscale Adv. 2024 Jul 19;6(18):4704–23. doi: 10.1039/d4na00363b (PMC11386170; doi:10.1039/d4na00363b)

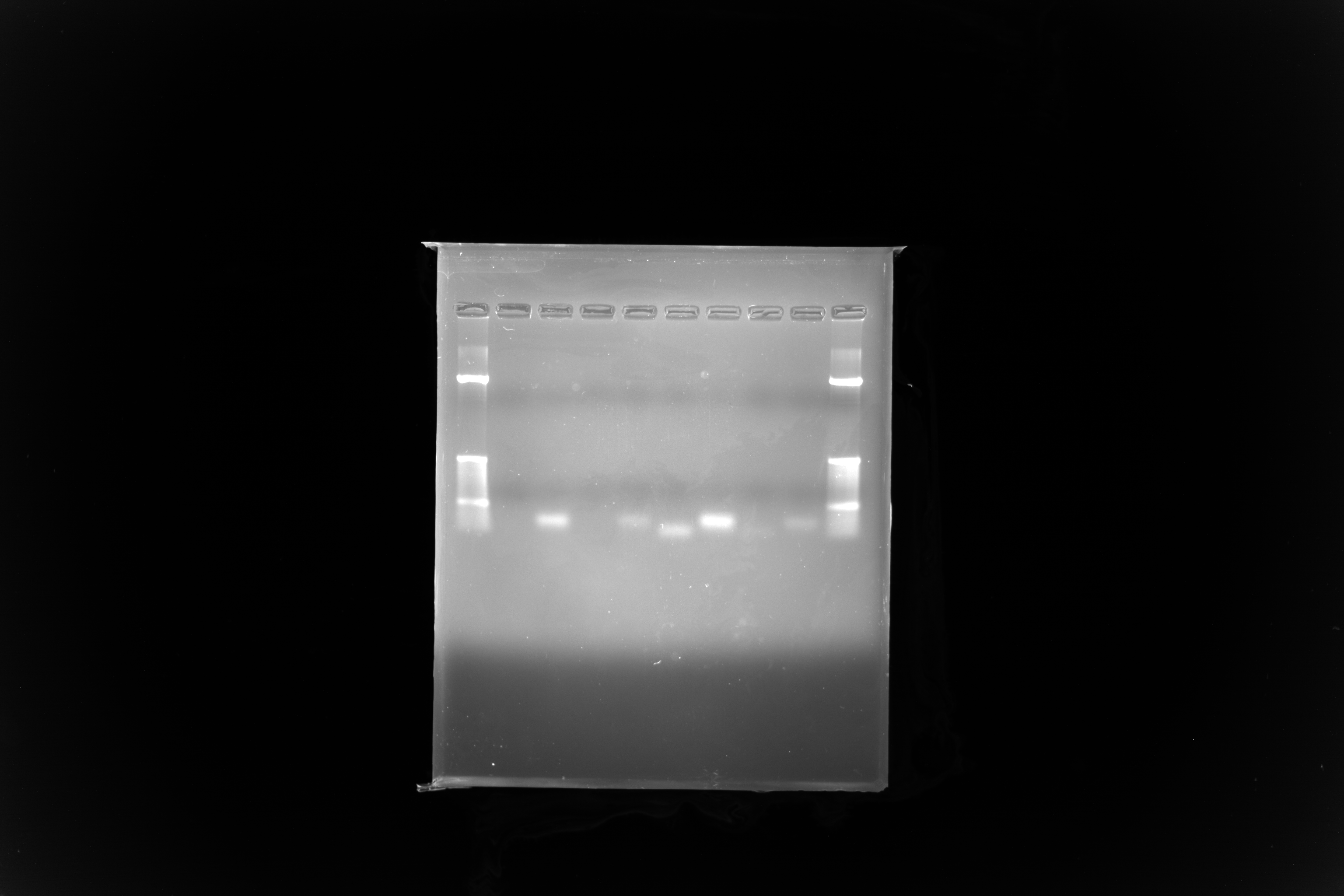

Supplement: NA-006-D4NA00363B-s002 [file NA-006-D4NA00363B-s002.zip › Rnase A_Controls.tif]

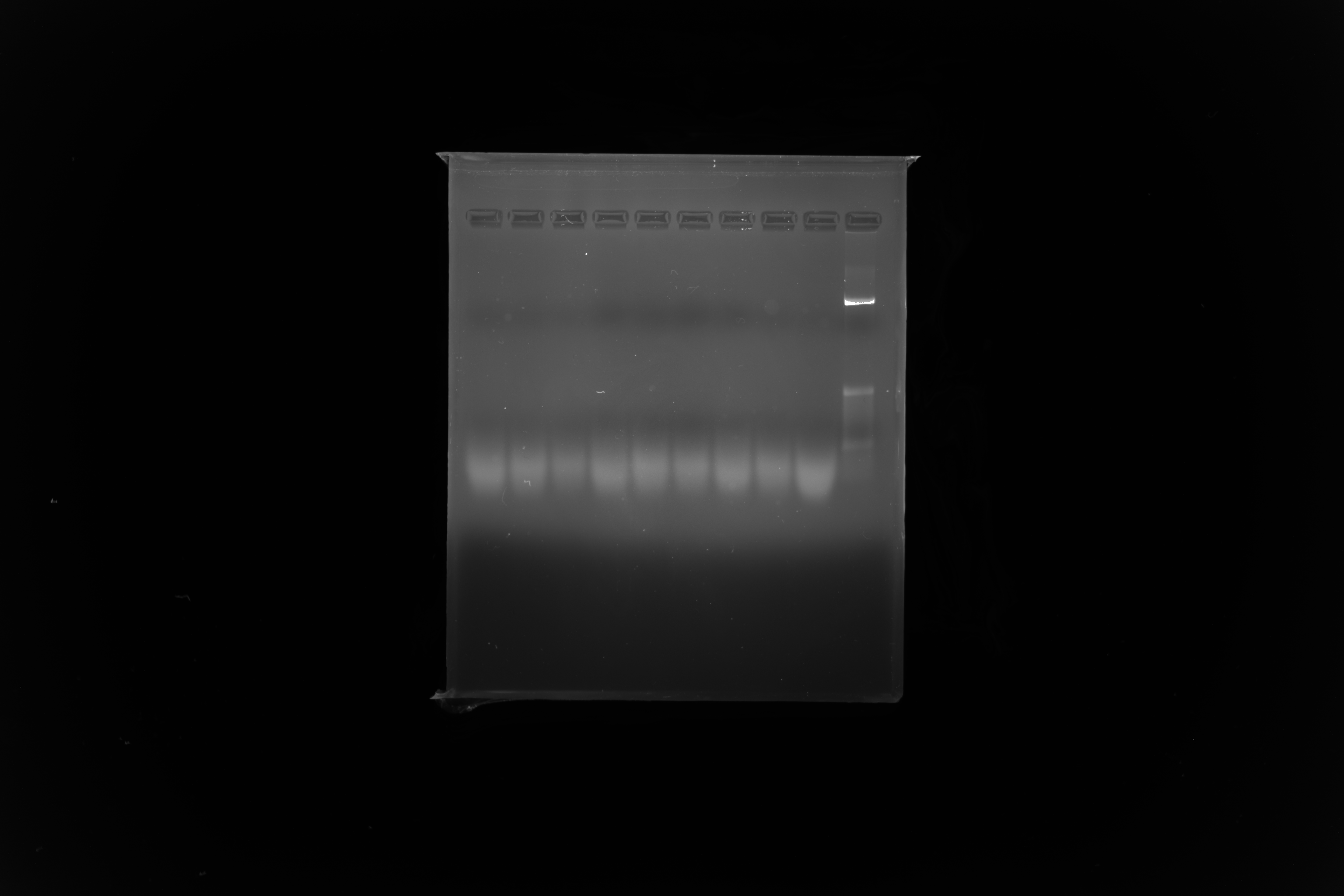

Supplement: NA-006-D4NA00363B-s002 [file NA-006-D4NA00363B-s002.zip › 4h_Rnase A assay.tif]

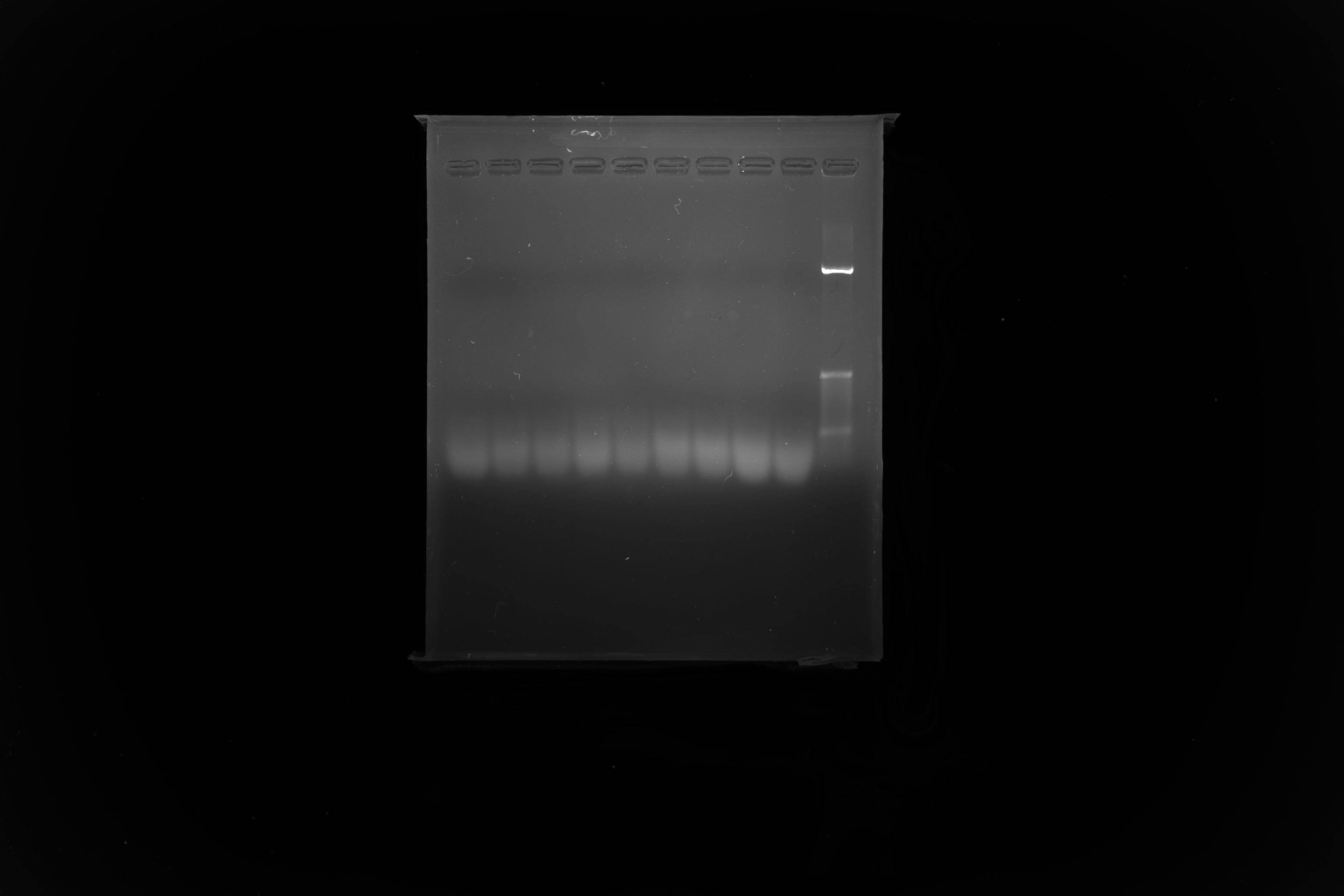

Supplement: NA-006-D4NA00363B-s002 [file NA-006-D4NA00363B-s002.zip › 24h_Rnase A assay.tif]

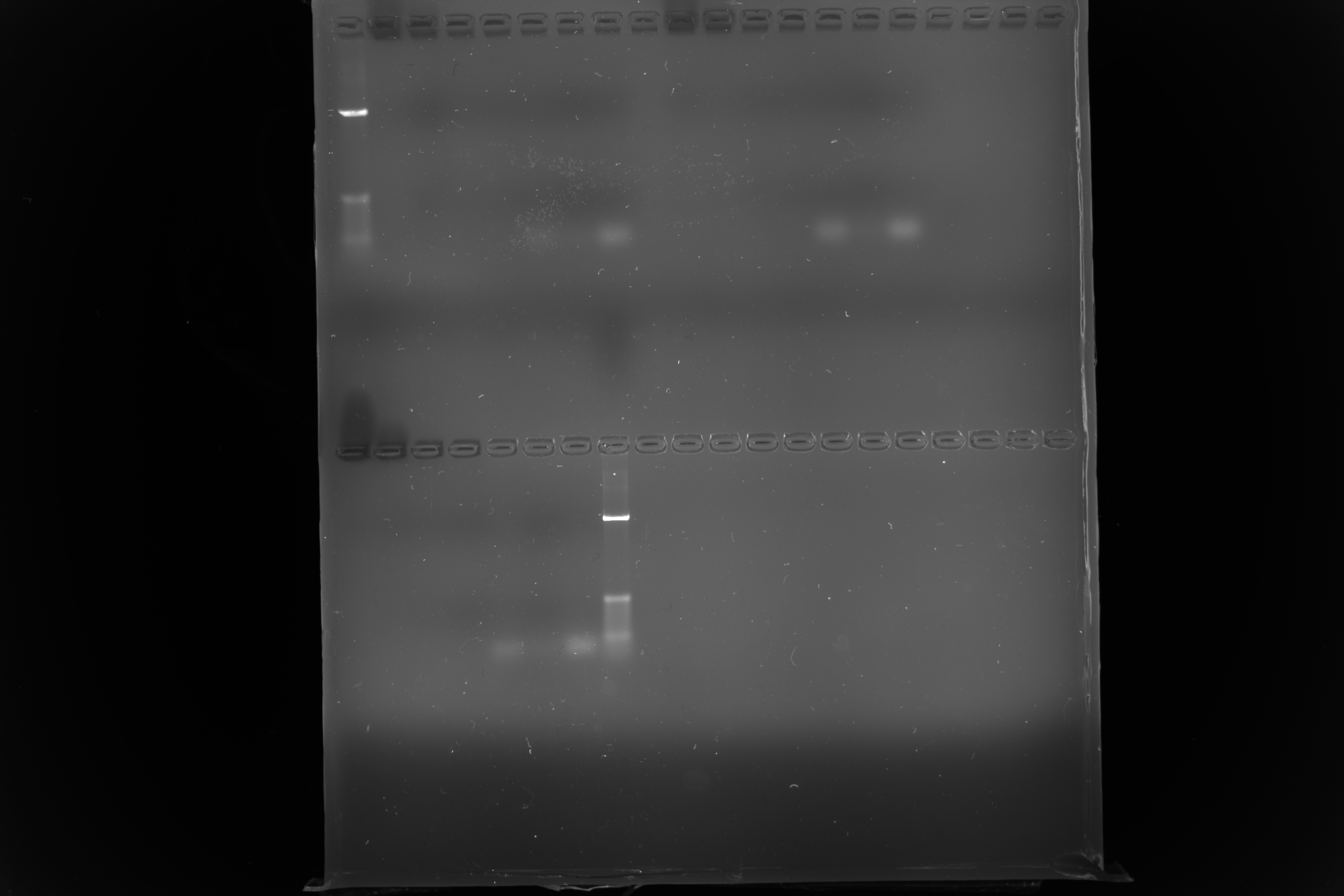

Supplement: NA-006-D4NA00363B-s002 [file NA-006-D4NA00363B-s002.zip › Dye exclusion assay_Figure 5c.tif]
